# Real-world implementation of a multilevel interventions program to prevent mother-to-child transmission of HBV in China

**DOI:** 10.1038/s41591-023-02782-x

**Published:** 2024-01-31

**Authors:** Xueru Yin, Wei Wang, Hui Chen, Qing Mao, Guorong Han, Lyufeng Yao, Qingwei Gao, Yunfei Gao, Jie Jin, Tong Sun, Minghua Qi, Hua Zhang, Baijun Li, Chongyang Duan, Fuqiang Cui, Weiming Tang, Polin Chan, Zhihua Liu, Jinlin Hou, SHIELD Study Group

**Affiliations:** 1 Department of Infectious Diseases, Nanfang Hospital, Southern Medical University, Guangzhou, China; 2 State Key Laboratory of Organ Failure Research; Key Laboratory of Infectious Diseases Research in South China, Ministry of Education; Guangdong Provincial Key Laboratory of Viral Hepatitis Research; Guangdong Provincial Clinical Research Center for Viral Hepatitis; Guangdong Institute of Hepatology, Guangzhou, China; 3 Department of Health Care, Shenzhen Bao’an Women’s and Children’s Hospital, Shenzhen, China; 4 Department of Hepatology, Hepatobiliary Hospital of Jilin, Changchun, China; 5 Department of Infectious Diseases, Southwest Hospital, Army Medical University, Chongqing, China; 6 Department of Gynecology and Obstetrics, The Second Affiliated Hospital of the Southeast University, Nanjing, China; 7 Department of Hepatology, Mengchao Hepatobiliary Hospital, Fujian Medical University, Fuzhou, China; 8 Department of Hepatology, The Sixth People’s Hospital of Dalian, Dalian, China; 9 Department of Gynecology and Obstetrics, Nanfang Hospital, Southern Medical University, Guangzhou, China; 10 Department of Infectious Disease, The First People’s Hospital of Hangzhou, Hangzhou, China; 11 Department of Hepatology, The Fifth People’s Hospital of Wuxi, Wuxi, China; 12 Department of Infectious Diseases, Peking University Shenzhen Hospital, Shenzhen, China; 13 Department of Gynecology and Obstetrics, Beijing Youan Hospital, Capital Medical University, Beijing, China; 14 Department of Hepatology, The Sixth People’s Hospital of Shenyang, Shenyang, China; 15 Department of Biostatistics, School of Public Health, Southern Medical University, Guangzhou, China; 16 School of Public Health, Peking University, Beijing, China; 17 Dermatology Hospital of South Medical University, Guangzhou, China; 18 University of North Carolina Project-China, Guangzhou, China; 19 Institute for Global Health and Infectious Diseases, University of North Carolina at Chapel Hill, Chapel Hill, NC USA; 20 World Health Organization South-East Asia Regional Office, New Delhi, India

**Keywords:** Hepatitis B, Epidemiology

## Abstract

Reducing hepatitis B virus (HBV) mother-to-child transmission (MTCT) is a fundamental step toward the HBV elimination goal. The multicentred, multilevel SHIELD program aimed to use an intense intervention package to reduce HBV MTCT in China. This study was conducted in diverse health settings across China, encompassing 30,109 pregnant women from 178 hospitals, part of the interim analysis of stage II of the SHIELD program, and 8,642 pregnant women from 160 community-level health facilities in stage III of the SHIELD program. The study found that the overall MTCT rate was 0.23% (39 of 16,908; 95% confidence interval (CI): 0.16–0.32%) in stage II and 0.23% (12 of 5,290; 95% CI: 0.12–0.40%) in stage III. The MTCT rate was lower among participants who were compliant with the interventions (stage II: 0.16% (95% CI: 0.10–0.26%); stage III: 0.03% (95% CI: 0.00–0.19%)) than among those who were noncompliant (3.16% (95% CI: 1.94–4.85%); 1.91% (95% CI: 0.83–3.73%); *P* < 0.001). Our findings demonstrate that the comprehensive interventions among HBV-infected pregnant women were feasible and effective in dramatically reducing MTCT.

## Abstract (web summary)

Interim analysis of the SHIELD program, adopting multilevel interventions, showcases low mother-to-child transmission (MTCT) rates of hepatitis B virus (HBV) across different hospital and community settings in China.

## Main

Approximately 17 people are newly infected with hepatitis B virus (HBV) every hour, primarily through mother-to-child transmission (MTCT), and nearly 1 million deaths are attributed to HBV annually globally^1,2^. China has the world’s largest hepatitis disease burden, with over 70 million people estimated to be living with HBV^3^. Moreover, ~23 million women of reproductive age are positive for the hepatitis B surface antigen (HBsAg), resulting in over 50,000 HBV-infected infants annually^4^. The World Health Organization (WHO) Global Health Sector Strategy on Viral Hepatitis calls for eliminating hepatitis B as a public health threat by 2030, with a reduction in the prevalence of HBsAg positivity to below 0.1% in children aged 5 years^5,6^. The global community has committed to eliminating HBV MTCT as a public health priority^6–8^. The Chinese government has also adopted this goal, and progress in preventing HBV MTCT in China should have a major impact on the global elimination of HBV by 2030.

The core components for preventing HBV MTCT in China include routine antenatal HBsAg screening in pregnant women^9^; prescribing additional antiviral drugs for HBsAg-positive women during pregnancy^1^ to reduce the risk of MTCT further^10–12^; ensuring the completion of the infant HBV vaccine series, with the timely administration of the hepatitis B vaccine birth dose (HepB-BD) and hepatitis B immunoglobulin (HBIG)^13,14^; and routine follow-up of HBV-exposed infants by postvaccination serological testing (PVST). Specifically, the Chinese National integrated prevention of mother-to-child transmission (iPMTCT) of human immunodeficiency virus (HIV), syphilis and hepatitis B program uses a coordinated approach toward achieving the elimination of HBV MTCT through access to quality reproductive, maternal, newborn and child health services for all women and their children in the context of universal health coverage^15^. In the iPMTCT program, free HBsAg testing is provided for all pregnant women and free immunoprophylaxis is provided for all infants born to HBsAg-positive women. On this basis, the SHIELD program is the first to integrate maternal antiviral therapy and PVST for HBV-exposed infants into the management algorithm for preventing HBV MTCT in real-world practice^16–18^.

Considering the large sample size and the potential impact these outcomes may have on global implementation programs aimed at eliminating HBV MTCT, this paper reports the interim outcomes of the SHIELD program.

## Results

### Baseline characteristics

**Fig. 1:**
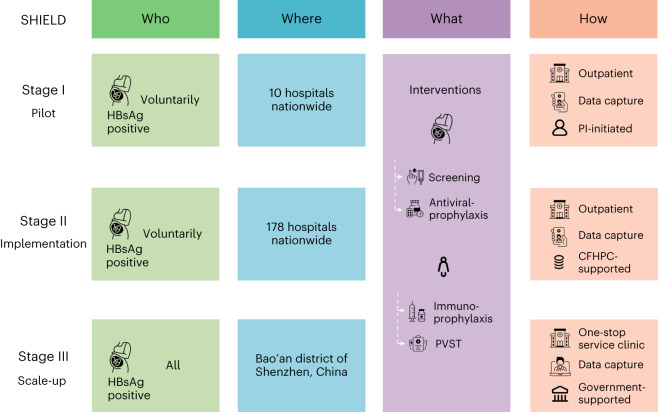
Schematic representation of the SHIELD program design. The SHIELD program comprised three stages. In stage I (July 2015–May 2018), which was a pilot stage to develop a management algorithm and tool for preventing HBV MTCT for stage II, 1,008 HBsAg-positive pregnant women at ten hospitals nationwide were voluntarily enrolled. In stage II (July 2015–December 2025), which was an implementation stage to apply the management algorithm and tool for preventing HBV MTCT from stage I in member hospitals across China, HBsAg-positive pregnant women at 178 hospitals nationwide were voluntarily enrolled. In stage III (January 2018–December 2019), the program was scaled up and implemented in all community health centers and hospitals in the Bao’an district of Shenzhen, China. One-stop services were especially established in Bao’an district to implement the intervention package for all pregnant women with HBV infection from 18 hospitals and 142 community health centers. PI, principal investigator.

Data from 30,109 pregnant women from the implementation stage (stage II; Extended Data Table 1) and 8,642 pregnant women from the scale-up stage (stage III) were included in this analysis (Fig. 1). The baseline characteristics are provided in Table 1. Overall, 53.51% of the mothers in the implementation stage and 24.86% of the mothers in the community scale-up stage had HBV DNA viral loads over 200,000 IU ml^−1^, while 57.36% and 25.36% of the mothers were hepatitis B e antigen (HBeAg)-positive, respectively. The majority of the mothers had normal alanine aminotransferase (ALT) levels in both stages. In stage II, 99.98% and 99.99% of exposed infants received timely HepB-BDs and HBIG, respectively, and 99.78% received the full three-dose HBV series. In stage III, 99.63% of exposed infants received timely HepB-BDs, 99.13% received timely HBIG and 99.59% received the full three-dose series of the HBV vaccine. The comparison of the baseline characteristics of the participants who were lost to follow-up and those who completed follow-up is provided in Extended Data Table 2.

**Table 1:** Characteristics of the mothers and infants

Characteristics	Implementation stage (*n* = 30,109)	Scale-up stage (*n* = 8,642)
Maternal characteristics		
Age (years)	28.36 ± 4.33	29.76 ± 4.51
Distribution of HBV DNA viral load (IU ml^−1^)		
Undetectable	23.31%	20.49%
Detectable to <200,000	23.17%	54.65%
≥200,000	53.51%	24.86%
HBeAg positivity	57.36%	25.36%
ALT (U l^−1^)	34.05 ± 63.10	18.09 ± 56.34
<40	83.16%	95.58%
≥40 to <80	9.80%	3.08%
≥80 to <200	5.11%	1.09%
≥200	1.93%	0.24%
Cesarean section	45.17%	33.71%
Infant characteristics		
Head circumference (cm)	33.66 ± 1.52	33.73 ± 1.26
Length (cm)	50.04 ± 2.08	49.76 ± 1.94
Birth weight (g)	3262.77 ± 531.17	3192.45 ± 472.41
Apgar score at 1 min	9.92 ± 0.40	9.81 ± 0.83
Timely HBIG administered	99.99%	99.13%
Timely HBV vaccine administered at birth	99.98%	99.63%
Full three-dose series of HBV vaccine administered	99.78%	99.59%

### HBV MTCT rates

**Fig. 2:**
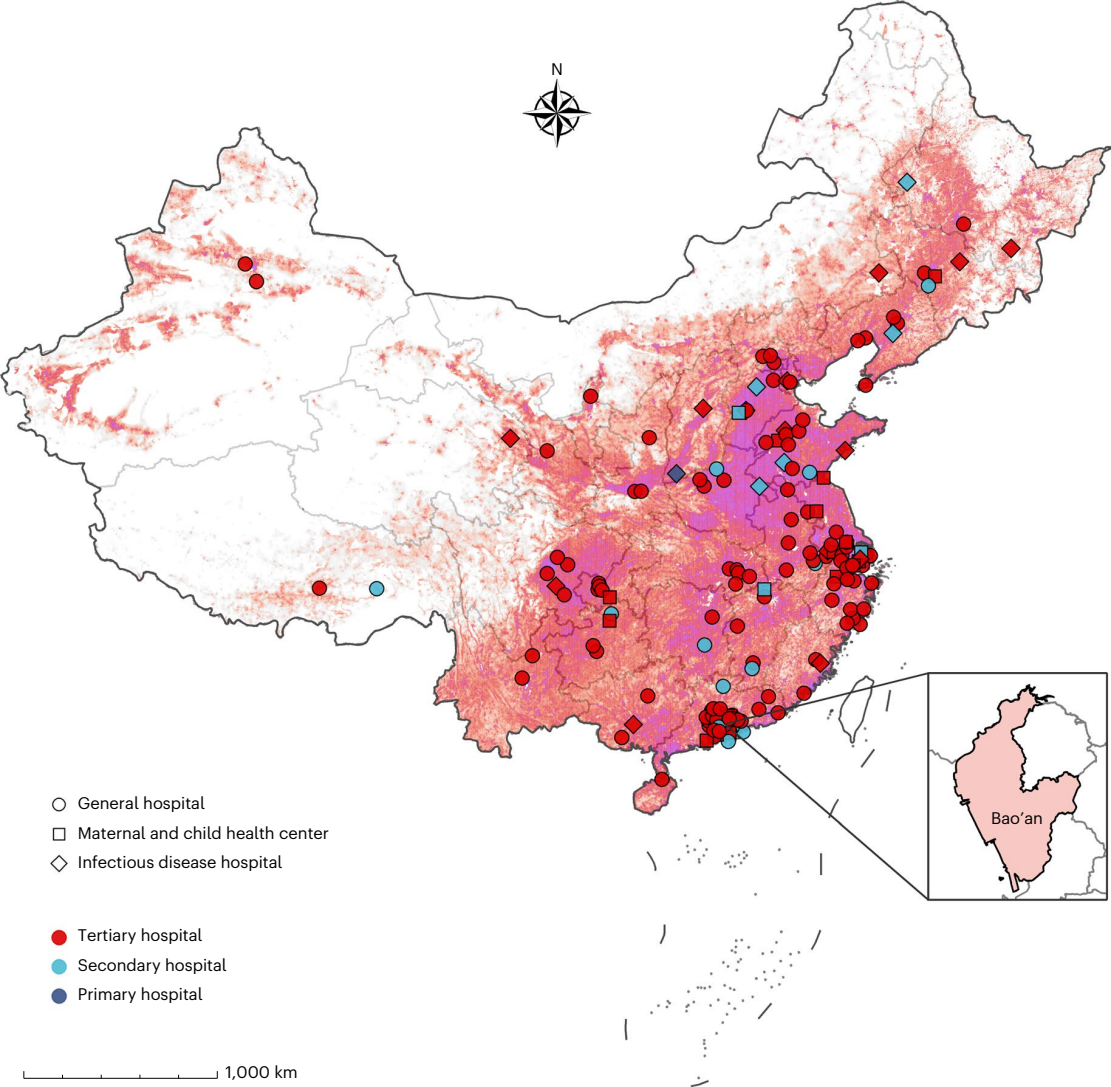
The geographical locations of the hospitals participating in the SHIELD program. In stage II, the SHIELD program consists of 178 member hospitals nationwide, including general hospitals, infectious disease hospitals, maternal and child health centers and tertiary, secondary and primary hospitals. In stage III, the program was implemented in Bao’an district of Shenzhen, China. A map of China is drawn by importing publicly released geographic data by the Ministry of Civil Affairs of the People’s Republic of China (http://xzqh.mca.gov.cn) and publicly released population density data by the WorldPop (https://www.worldpop.org) into R software. The fall colors in the map represent the population density, ranging from low (light red) to high (purple).

**Fig. 3:**
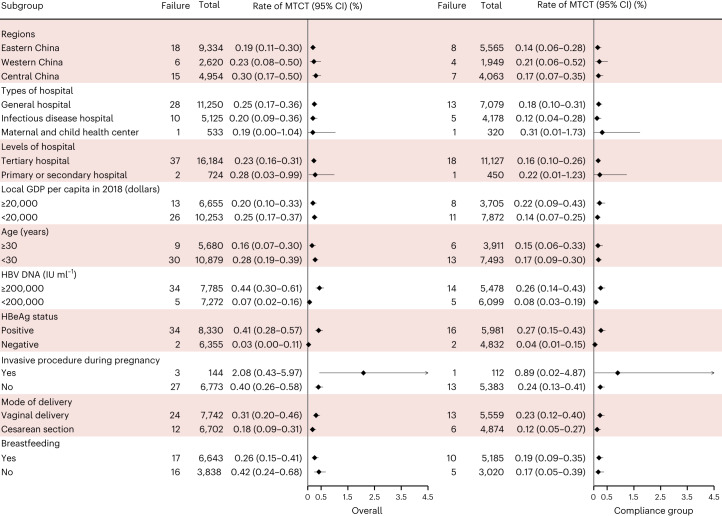
Forest plot of HBV MTCT rate in the subgroups in the implementation stage of the SHIELD program. The HBV MTCT rates were stratified by sociodemographic characteristics and the compliance group. Pregnant women from 31 provinces were divided into the following three regions according to the addresses of hospitals they visited to analyze regional disparities: eastern China, central China and western China. Compliance refers to patients’ compliance with the whole process of HBV mother-to-child prevention management strictly by the following two situations: (1) for patients with HBV DNA ≥200,000 IU ml^−1^ (defined as a high-risk group), antiviral therapy was initiated at 24–28 weeks gestation, and their newborn completed immunization (including birth dose of HepB and HBIG within 12 h, and the completion of three doses of HepB); (2) for patients with HBV DNA <200,000 IU ml^−1^ (defined as a low-risk group), their newborns were immunized (including birth dose of HepB and HBIG) within 12 h and completed three doses of HepB. The data are presented as rates (points) and 95% CIs (error bars).

**Extended Data Fig. 1:**
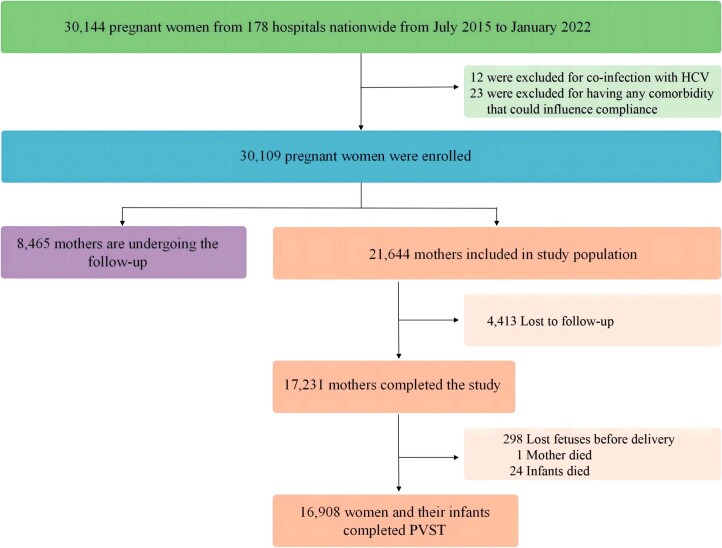
Cohort selection in the implementation stage of SHIELD. In the implementation stage, 30,109 pregnant women were enrolled. Furthermore, 8,465 underwent follow-up, and 21,644 pregnant women were included in study population. Finally, PVST was completed for 16,908 participants. PVST, postvaccination serological testing.

In the implementation stage, PVST was completed for 16,908 participants, and 8,465 underwent follow-up (the flow chart is shown in Extended Data Fig. 1). To date the overall MTCT rate was 0.23% (39 of the 16,908 pregnant women with HBV infection; 95% CI: 0.16–0.32%) and lost to follow-up rate for those who completed follow-up was 20.39% (4,413 of 21,644 participants). We also analyzed the MTCT rates by regions, types of hospital, levels of hospital, local gross domestic product per capita, age, HBV DNA viral load, HBeAg status, invasive procedures during pregnancy, mode of delivery and breastfeeding (Figs. 2 and 3).

**Extended Data Fig. 2:**
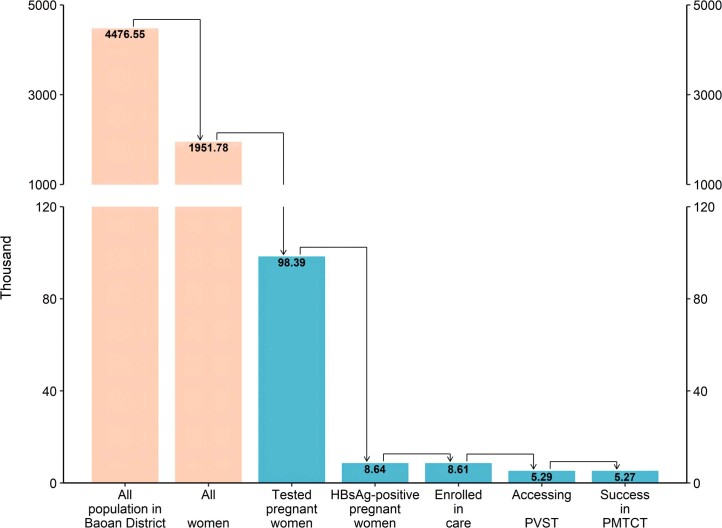
The prevention of mother-to-child transmission of hepatitis B virus service cascade in the scale-up stage of SHIELD. The population in Bao’an district was 4,476,554, and there were 1,951,778 women. 98,391 pregnant women received HBsAg testing, and 8643 pregnant women were HBsAg positive. 8610 HBsAg-positive pregnant women enrolled in care, of which 5290 completed PVST and 5269 succeeded in preventing MTCT. The fill colors of bar represent data sources from official statistics of the seventh Shenzhen census (light pink) (http://www.sz.gov.cn/zfgb/2021/gb1199/content/post_8806392.html) and SHIELD (blue). HBsAg, hepatitis B surface antigen; PVST, postvaccination serological testing; PMTCT, prevention of mother-to-child transmission. (http://www.sz.gov.cn/zfgb/2021/gb1199/content/post_8806392.html) and SHIELD (blue).

In the community scale-up stage, 98,391 pregnant women were screened for HIV, syphilis and HBV, and 8,643 tested positive for HBsAg in Shenzhen Bao’an district. The overall MTCT rate was 0.23% (12 of 5,290 pregnant women with HBV infection; 95% CI: 0.12–0.40%; Extended Data Fig. 2).

### Rate of birth defect

In the community scale-up stage, the rate of birth defect was 1.68% (145 of 8,642; 95% CI: 1.42–1.97%). There was no difference in birth defect rates between participants who received antiviral interventions (1.19%; 11 of 921 participants; 95% CI: 0.60–2.13%) versus those who did not (1.74%; 134 of 7,721 participants; 95% CI: 1.46–2.05%; *P* = 0.277).

### Post hoc analysis

**Fig. 4:**
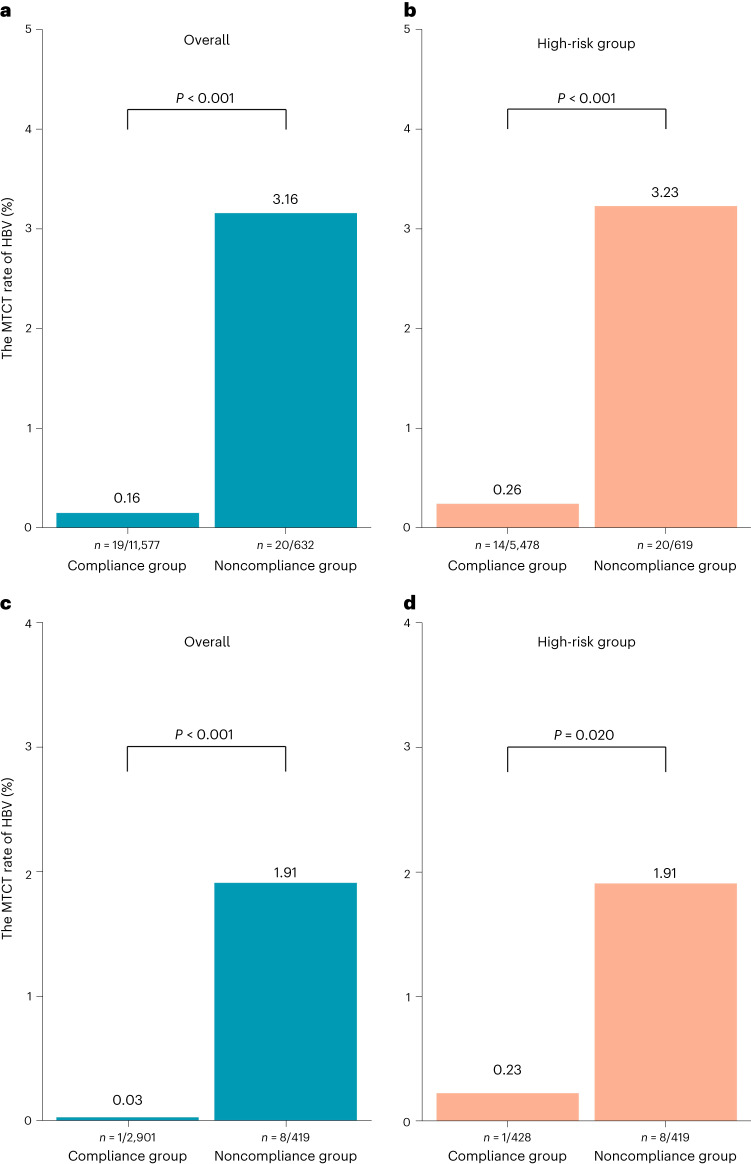
The HBV MTCT rates in compliance and noncompliance groups. **a**–**d**, The HBV MTCT rate in the overall and high-risk groups in the implementation stage (**a**,**b**) and in the scale-up stage (**c**,**d**) of the SHIELD program. **a**, In the implementation stage, the overall MTCT rate was 0.16% (19 of 11,577; 95% CI: 0.10–0.26%) among compliant participants and 3.16% (20 of 632; 95% CI: 1.94–4.85%) among noncompliant participants (*P* < 0.001). **b**, In the implementation stage, the MTCT rate in the high-risk group was 0.26% (14 of 5,478; 95% CI: 0.14–0.43%) among compliant participants and 3.23% (20 of 619; 95% CI: 1.98–4.95%) among noncompliant participants (*P* < 0.001). **c**, In the community scale-up stage, the MTCT rate was 0.03% (1 of 2,901; 95% CI: 0.00–0.19%) among compliant participants and 1.91% (8 of 419; 95% CI: 0.83–3.73%) among noncompliant participants (*P* < 0.001). **d**, In the community scale-up stage, the MTCT rate in the high-risk group was 0.23% (1 of 428; 95% CI: 0.01–1.29%) among compliant participants and 1.91% (8 of 419; 95% CI: 0.83–3.73%) among noncompliant participants (*P* = 0.020). Compliance refers to patients’ compliance with the whole process of HBV mother-to-child prevention management strictly by the following two situations: (1) for patients with HBV DNA ≥200,000 IU ml^−1^ (defined as a high-risk group), antiviral therapy was initiated at 24–28 weeks gestation, and their newborn completed immunization (including a birth dose of HepB and HBIG within 12 h, and the completion of three doses of HepB); (2) for patients with HBV DNA <200,000 IU ml^−1^ (defined as low-risk group), their newborns were immunized (including birth dose of HepB and HBIG) within 12 h and completed three doses of HepB. Categorical variables were analyzed with Pearson’s chi-square tests. *P* values are two-sided.

In the implementation stage, the overall MTCT rate was 0.16% (19 of 11,577 participants; 95% CI: 0.10–0.26%) among participants who complied with the comprehensive interventions and 3.16% (20 of 632 participants; 95% CI: 1.94–4.85%) among noncompliant participants (Fig. 4a). Among noncompliant participants with HBV DNA viral loads ≥200,000 IU ml^−1^, 606 mothers did not receive antiviral treatment, two infants did not receive timely HepB-BDs and 11 infants did not receive the full three-dose series of the HBV vaccine. Among noncompliant participants with HBV DNA viral loads <200,000 IU ml^−1^, one infant did not receive HBIG on time, one infant did not receive a timely HepB-BD and 11 infants did not receive the full three-dose series of the HBV vaccine.

Subgroup analysis showed an MTCT rate of 0.26% (14 of 5,478 participants; 95% CI: 0.14–0.43%) among compliant participants in the high-risk group (defined by a maternal HBV DNA viral load ≥200,000 IU ml^−1^) and 3.23% (20 of 619 participants; 95% CI: 1.98–4.95%) among noncompliant participants (Fig. 4b).

The multivariate analysis results indicated that not receiving an antiviral treatment intervention, HBeAg positivity, an HBV DNA viral load ≥200,000 IU ml^−1^ and undergoing invasive procedures during pregnancy were associated with HBV MTCT (Extended Data Table 3).

In the community scale-up stage, the MTCT rate was significantly lower among the participants who complied with the comprehensive interventions (0.03%; 1 of 2,901 participants; 95% CI: 0.00–0.19%) than noncompliant (1.91%; 8 of 419 participants; 95% CI: 0.83–3.73%; *P* < 0.001; Fig. 4c). Among participants whose compliance with the comprehensive interventions could not be assessed due to of missing information, the MTCT rate was 0.15% (3 of 1,970 participants; 95% CI: 0.03–0.44%).

For participants with an HBV DNA viral load ≥200,000 IU ml^−1^, the MTCT rates were 0.23% (1 of 428 participants; 95% CI: 0.01–1.29%) among participants who were compliant with the comprehensive interventions compared to 1.91% (8 of 419 participants; 95% CI: 0.83–3.73%) among noncompliant participants (*P* = 0.020; Fig. 4d). Notably, all participants with an HBV DNA viral load ≥200,000 IU ml^−1^ who did not comply with the comprehensive interventions did not receive antiviral treatment.

## Discussion

Preventing HBV MTCT is critical for achieving the global goal of eliminating viral hepatitis by 2030. Evaluating the effectiveness of ongoing elimination programs is essential to tailoring the programs further to achieve the goal. Findings from our SHIELD study extend the existing literature by evaluating setting effects, summarizing evidence across different economic levels of China and assessing the program’s impact in real-world settings. Overall, for both stages II and III of the SHIELD program, the MTCT rate was low in both the hospital and community settings and remained consistently low across other settings. Thus, implementing comprehensive interventions like SHIELD could help reduce HBV MTCT rates regardless of settings.

The overall MTCT rate in this study was as low as 0.23% in the implementation and community scale-up stages. This rate is substantially lower than the HBV MTCT rate of 1.40–2.00% reported by recent publications with only immunoprophylaxis^19–23^. Our study reports the HBV MTCT rate based on a large-scale sample in the era of antiviral prophylaxis use in real-world settings. Many factors may have contributed to the observed low MTCT rate that was observed in such a setting. First, China has achieved and exceeded the WHO Western Pacific region target for HBV vaccination coverage. In mainland China, universal HBV vaccination in newborns started in 1992, and the vaccine has been provided free of charge since 2002. Vaccination services for newborns have also been free since 2005. In addition, the iPMTCT program covered all pregnant women in 2015, providing free HBsAg testing and free HBIG for infants born to HBsAg-positive mothers. Thus, the coverage of birth-dose HBV vaccine and HBIG nearly reached 100% in the SHIELD study, although global coverage of HBV birth-dose vaccination coverage in 2015 remained low at 38%^6^. Second, providing a timely birth dose of the HBV vaccine and HBIG in a timely manner is key to preventing HBV MTCT. In the SHIELD study, most newborns received a HepB-BD and HBIG within 2 h after birth. Third, the proportion of women with antiviral treatment compliance during pregnancy was as high as 83.2% in mothers with high viral loads. This may be because antivirals are accessible in most hospitals in China, and the price of tenofovir disoproxil fumarate (TDF) for treating HBV has been dramatically reduced in mainland China through government negotiations (under US $1.45 per month). Thus, the cost of antiviral therapy is affordable for most patients.

We found that the rates of HBV MTCT remained consistent across various geographic, socioeconomic status and hospital settings. This implies that the intervention implemented in our study could be feasible and effective across diverse settings, including western China (remote area), low- and middle-income areas and primary and secondary hospitals. Besides that, antivirals and HepB are widely available worldwide, and the implementation algorithm is easy to follow or tailor for varied settings. Furthermore, the wide application of mobile internet services makes it possible for digital applications to be adapted as health management tools. Therefore, HBV MTCT prevention services should be expanded and decentralized to facilitate the achievement of the HBV elimination goal. This finding would help direct global actions to generalize comprehensive interventions, especially in low- and middle-income countries.

We also found that the HBV MTCT rate was 0.16% in the hospital setting and 0.03% in the community setting among participants managed under the current comprehensive interventions. These rates were substantially lower in compliant participants than in those who were not. The same trend was observed among participants with an HBV DNA viral load ≥200,000 IU ml^−1^. This finding is consistent with previous small sample studies^10–12^ and demonstrates the effectiveness of the current comprehensive interventions. However, our findings also showed that antiviral treatment compliance in mothers with an HBV DNA viral load ≥200,000 IU ml^−1^ may be a vulnerable area for implementing comprehensive interventions.

According to the results, not receiving antiviral therapy, being HBeAg-positive, having an HBV DNA viral load ≥200,000 IU ml^−1^ and undergoing invasive procedures during pregnancy were associated with increased risk of HBV MTCT. This finding concurs with previous studies^17,18,24,25^, indicating that HBeAg-positive pregnant women with high HBV DNA levels have an increased risk for HBV MTCT and should be paid more attention. Additionally, invasive procedures among HBeAg-positive pregnant women during pregnancy increase the risk of HBV transmission to the child. Therefore, the need for invasive procedures during pregnancy should be carefully evaluated by obstetricians. Antiviral intervention should be administered in advance as per the clinical practice of PMTCT to reduce transmission risk if an invasive procedure is required.

The SHIELD program is a vital complement to the existing government PMTCT program for the following reasons: (1) in real-world practice, the SHIELD program integrated HBV DNA viral load, HBeAg, maternal antiviral intervention and HBV-exposed infant PVST into the management algorithm for preventing HBV MTCT since 2015 and the iPMTCT program integrated those nationwide since 2022; (2) the SHIELD program developed an app to facilitate patient management; (3) the SHIELD program explored a one-stop service for the management of PMTCT of HBV, which has a great potential to improve treatment uptake and compliance. This has been adopted by the government (Supplementary Fig. 1).

As a large-scale long-term study, our study has several important implications. First, the health administration should make policies to provide a foundation for eliminating MTCT. Immunization and iPMTCT programs have laid the groundwork for the success of preventing HBV MTCT. Screening, treatment and vaccination services for HBV are offered during antenatal, delivery and postnatal care. PVST should be included in essential health service packages, with access ensured and covered by public funding. Second, implementing comprehensive interventions on a large scale is feasible and effective. The SHIELD study findings prove that comprehensive interventions could dramatically reduce the HBV MTCT rate to 0.16% in the hospital setting and 0.03% in the community setting, which is much lower than the WHO recommended goal of 2%^26^. Third, it is important to define a core package of evidence-based interventions along the service continuum that is relevant to each country’s context and tailored to the needs of diverse populations and settings. Data, scientific evidence, good practice, community input, HBV disease burden, equity, effectiveness, cost, acceptability and feasibility should inform the selection of such interventions. For example, a standardized management algorithm was lacking at the beginning of the SHIELD program; thus, experts in the fields of infectious diseases, hepatology, immunology, obstetrics and public health were recruited to develop an algorithm for preventing MTCT in clinical practice in China^18^. In addition, eliminating HBV requires close collaboration among stakeholders, including the government, nongovernmental organizations, medical communities, health providers and patients. For example, the Chinese Foundation for Hepatitis Prevention and Control (CFHPC), which is a national-level public welfare foundation with strong social influence, had a substantial role in service delivery and advocacy and was responsible for training all medical personnel involved in stage II of the SHIELD program. Furthermore, data capture and participant management could be a great challenge for projects with large-scale samples and limited funding. There are great challenges regarding data capture and participant management. Tools such as the SHIELD app improve work efficiency and increase treatment compliance, as participants can upload laboratory test reports and consult with their doctors without time or location restrictions.

Briefly speaking, the SHIELD program provided an example of accelerating the elimination of MTCT of HBV. There are three essential elements for implementing PMTCT. First, the management algorithm for preventing HBV MTCT should be standardized according to international consensus and be applied in the practice of PMTCT. Second, digital health could be used as a management tool to follow-up with participants and collect data. Third, the implementation process can be rolled out in multiple stages, including pilot, implementation and community scale-up stages, regardless of geographic, socioeconomic status and hospital settings.

Our study has some limitations. First, there were potential selection biases in the implementation stage, as not all participants were contacted by the listed hospitals enrolled in the study. Instead, convenience sampling was used to recruit participants, and HBeAg-positive women and those with a high-level HBV DNA were more willing to participate in the SHIELD program. However, we contemplated that the selection biases would not limit our results, as all the HBV-positive women identified were enrolled in the community scale-up stage. Additionally, the transmission rates were consistent in the implementation and community scale-up stages, and the rates of MTCT remained consistent across settings of varied geographic distributions, socioeconomic status and hospital/community levels. Second, some patients incurred out-of-pocket costs, as antivirals and testing in the SHIELD program were not completely free, which may have affected antiviral therapy compliance. However, these results may be more relevant as they depict real-world situations. Third, some participants were lost to follow-up, and the baseline characteristics were substantially different between the participants who were lost to follow-up and those who completed follow-up in stage II, which may induce selection bias on the outcomes. However, we believe that the influence of drop-outs was minor because (1) the baseline characteristics in the community scale-up stage were comparable between those who dropped out and those who completed the study, and the MTCT rates were similar to that in the implementation stage; (2) the MTCT rates were not substantially altered due to the large sample size, and the low rates remained salient across various settings during the implementation stage; (3) we recruited a higher proportion of HBeAg-positive pregnant women and those with a high level of HBV DNA in the cohort of stage II; thus, the MTCT rate might not be underestimated. We aimed to assess whether MTCT rates would decrease by adopting the package of comprehensive interventions. Therefore, regarding the MTCT rate, the results are reliable and were least altered by the dropout. As the main reason for the dropout was that some participants were a floating population who usually returned to their hometown after delivery, our findings remain salient. Besides that, we did not report the birth defect rates in stage II, because the data source is still undergoing verification by the China Birth Defect Monitoring Center per protocol pending completion of stage II implementation. In this study, we only report stage III birth defect rates as the data source for this stage stands verified. Furthermore, the analysis in stage III showed that the birth defect rate was similar among the participants who received antiviral intervention and those who did not, and we expect that the birth defect rates will be similar between stages II and III.

In conclusion, the SHIELD study provides successful pragmatic experience and real-world evidence for implementing comprehensive interventions for preventing HBV MTCT. Our findings showed that the comprehensive intervention resulted in low MTCT rates across different settings. This offers a great example of generalizing interventions for the national and global elimination of HBV MTCT.

## Methods

### Overview of the SHIELD program

The SHIELD program aims to accelerate the elimination of HBV MTCT. The program comprised three stages and was launched in China in 2015, as shown in Fig. 1. In this study, we mainly present the findings of an interim analysis of stage II and the final analysis of stage III to study the effectiveness in the prevention of HBV MTCT with the SHIELD program.

In stage I (July 2015–May 2018; ClinicalTrials.gov registration: NCT03539016), which was a pilot stage to develop a management algorithm and tool for preventing HBV MTCT for stage II, 1,008 HBsAg-positive pregnant women at ten hospitals nationwide were enrolled, and the overall MTCT rate was 0.88%, as previously reported^17^. More importantly, based on the pilot stage, a management algorithm for preventing HBV MTCT was developed and further implemented in stages II and III^18^. At the beginning of the SHIELD program, a standardized management algorithm for preventing HBV MTCT in China was lacking, especially regarding various antiviral practices; thus, experts in the fields of infectious diseases, hepatology, immunology, obstetrics and public health were recruited by the SHIELD program to adapt the algorithm for preventing MTCT to clinical practice in China. This was the first technical guidance on the management preventing HBV MTCT in China.

In stage II (July 2015–December 2025; this interim analysis: July 2015–January 2022, ClinicalTrials.gov registration: NCT05172453), which was an implementation stage to scale up the management algorithm and tool for preventing HBV MTCT from stage I, comprehensive interventions for preventing HBV MTCT were implemented in the hospital setting across China in 2015, which covered hospitals in 31 provinces, municipalities and autonomous regions (Fig. 2). The SHIELD program established management committee responsible for management regulation, setting goals, mobilization and supervision, as well as Academic Committee responsible for study design, data analysis, academic exchange and real-world study. The SHIELD program consists of ten centers of excellence (COEs) and 178 member hospitals nationwide, including general hospitals, infectious disease hospitals, maternal and child health centers and tertiary, secondary and primary hospitals. Ten hospitals were chosen as COEs due to their experiences and skills in the field of PMTCT, and the COEs were responsible for technical guidance, transfer treatment, training the medical staff from nearby member hospitals on algorithm and app and ensuring that the management algorithm was correctly followed. The eligibility criteria included pregnant women who had been HBsAg-positive for over 6 months. Pregnant women were excluded if they had a positive serological test for HIV or hepatitis C virus or had any comorbidity that could influence compliance. In our study, pregnant women who did not have a smartphone and did not want to use the app were excluded because we obtained data through the app.

In stage III (January 2018–December 2019; ClinicalTrials.gov registration: NCT05172453), the program was scaled up and implemented in all community health centers and hospitals (hospital-community integrated strategy) in the Bao’an district of Shenzhen, which has a population of 4.48 million people. The Bao’an district was chosen for community scale-up because of the following reasons: (1) it has good representativeness with an HBsAg prevalence rate of 8.79% among pregnant women. Furthermore, immigrants account for 10.04% of the population^27^; (2) it was designated as one of the six pilot areas for the elimination of MTCT in China by the government in September 2017; therefore, strong policies could support the implementation of the intervention package among all pregnant women with HBV infection and (3) it has a SHIELD technical team appointed by the government, resulting in the provision of interventions in the implementation stage. One-stop services were especially established in Bao’an district to implement the intervention package for all pregnant women with HBV infection from 18 hospitals and 142 community health centers between January 2018 and December 2019 (Supplementary Fig. 2).

### Comprehensive interventions for preventing HBV MTCT

The management algorithm for preventing HBV MTCT is shown in Supplementary Fig. 3 and Supplementary Note. The sex of the participant was self-reported. In summary, antenatal HBsAg testing is universally and routinely offered to all pregnant women based on the iPMTCT program in China. Pregnant women with positive antenatal HBsAg tests are linked to appropriate clinical care services for managing chronic HBV infection^18^. The HBV infection status of the enrolled pregnant women was assessed at baseline. Pregnant women with evidence of cirrhosis or significant hepatic activity with an ALT level ≥5 times the upper limit of normal were started on long-term antiviral treatment. Pregnant women with no evidence of cirrhosis or significant hepatic activity were monitored at least every 4 weeks during pregnancy and were administered antiviral treatment if indicated. Pregnant women who did not require antiviral treatment but had a high HBV DNA viral load (the eligibility criterion was ≥200,000 IU ml^−1^) were considered at increased risk of HBV MTCT. Maternal antiviral prophylaxis, using TDF or telbivudine (LdT), was initiated at 24–28 weeks gestation and continued until delivery. Infants received the HepB-BDs (10 μg/0.5 ml) and HBIG (100 IU) as soon as possible after delivery (within 12 h). The other two HBV vaccine doses (10 μg/0.5 ml) were scheduled at 1 and 6 months of age following the national Chinese vaccination schedule. PVST was performed after the completion of the HBV vaccine series and at least 1 month after the last HBV vaccine dose (at ages 7–12 months). HBV infection was defined as being seropositive for HBsAg.

### SHIELD application

A mobile health application called the ‘SHIELD app’ was developed (Supplementary Fig. 4). Participants could consult with their doctors for free via the SHIELD app during follow-up. The doctors trained the participants on how to use the SHIELD app at baseline. During follow-up, the participants captured all laboratory test reports as pictures and uploaded them into the SHIELD app. The doctors or research assistants uploaded additional information into the SHIELD app. All participant information was prospectively collected through the SHIELD app (before the PVST outcome was known).

The data management group was responsible for converting the data uploaded to the SHIELD app to digital information. Ten percent of laboratory test reports were routinely monitored every week. Missing data were retrieved via the SHIELD app and telephone follow-up.

### Ethics

This study was conducted following the guidelines of the Declaration of Helsinki and the principles of good clinical practice and was approved by the Nanfang Hospital Ethics Committee. Electronic informed consent was obtained via the SHIELD app.

### Outcomes

Primary outcomes were the rate of MTCT of HBV and the rate of birth defects. MTCT of HBV was defined as being seropositive for HBsAg of infants after completing PVST. Birth defects were diagnosed by the obstetricians referred to the national standards for medical diagnosis of birth defects.

### Statistical analysis

Continuous variables are reported as the mean (±s.d.), and categorical variables are presented as percentages. Categorical variables were analyzed with Pearson’s chi-square or Fisher’s exact tests. Univariate logistic regression analysis was used to identify clinically relevant variables associated with MTCT in stage II. Collinearity diagnostics were conducted, and variables with a *P* < 0.10 in the univariable logistic regression analysis were entered into the multivariable logistic regression model. All statistical tests were two-sided. A *P* < 0.05 was considered to indicate statistical significance. All data were analyzed using R software 2022, version 4.2.2.

We performed sample size estimation for the post hoc analyses. The sample proportion is assumed to be 0.02. To produce a confidence interval with a width of no more than 0.005, 12,047 subjects were needed. Assuming the dropout rate was 20%, 15,059 subjects will be needed (sample size was computed using PASS 2022, version 22.0.3.). Thus, although the study has not been finished yet, the sample size was large enough for this interim analysis.

### Reporting summary

Further information on research design is available in the Nature Portfolio Reporting Summary linked to this article.

## Online content

Any methods, additional references, Nature Portfolio reporting summaries, source data, extended data, supplementary information, acknowledgements, peer review information; details of author contributions and competing interests; and statements of data and code availability are available at 10.1038/s41591-023-02782-x.

### Supplementary information

Supplementary Information Supplementary SHIELD Study Investigators, Figs. 1–4, Note and research protocol.

Reporting Summary

## Figures and Tables

**Extended Data Table 1:**
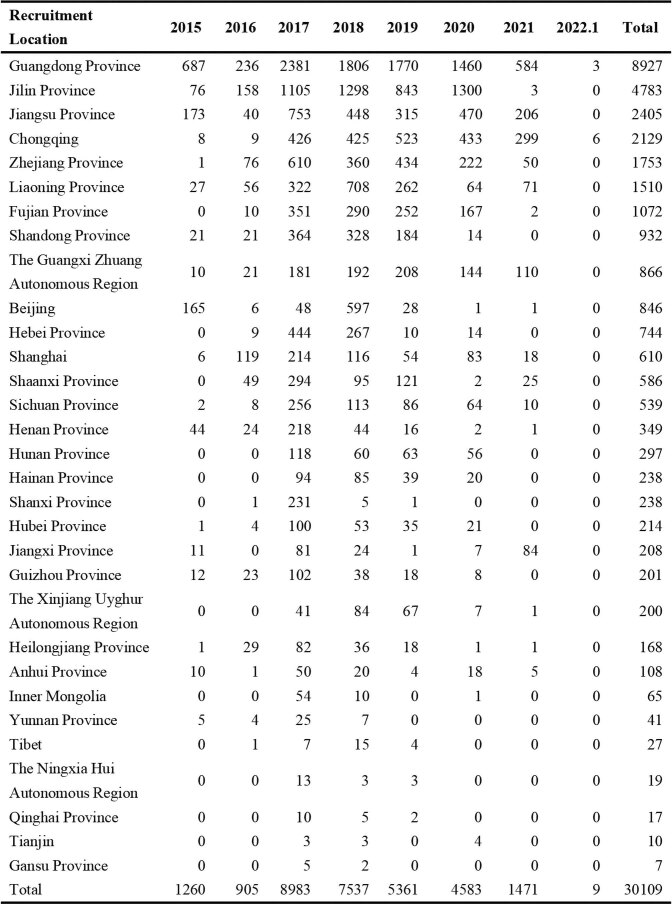
Recruitment location and year in the implementation stage of SHIELD

A total of 30,109 pregnant women were enrolled from 31 provinces, municipalities and autonomous regions during 2015 to January 2022.

**Extended Data Table 2:**
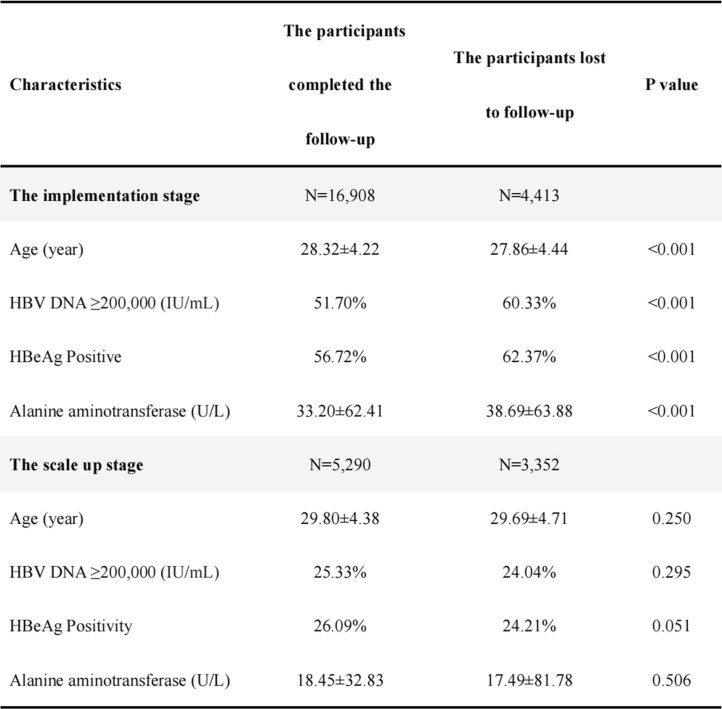
Comparison of the baseline characteristics of the participants lost to follow-up and the participants who completed the follow-up in the implementation stage and scale-up stage of SHIELD

We compared the baseline characteristics of the participants who lost to follow-up and the participants who completed the follow-up in the implementation stage and scale up stage of SHIELD. Categorical variables were analyzed with Pearson’s chi-square tests. Continuous variables were analyzed with independent-sample t-tests. P values are two-sided. A p value < 0.05 was considered to indicate statistical significance (age (p < 0.001); HBV DNA ≥ 200,000 IU/mL (p < 0.001); HBeAg-positive (p < 0.001); alanine aminotransferase (p < 0.001) in the implementation stage). HBeAg, hepatitis B e antigen; HBV, hepatitis B virus.

**Extended Data Table 3:**
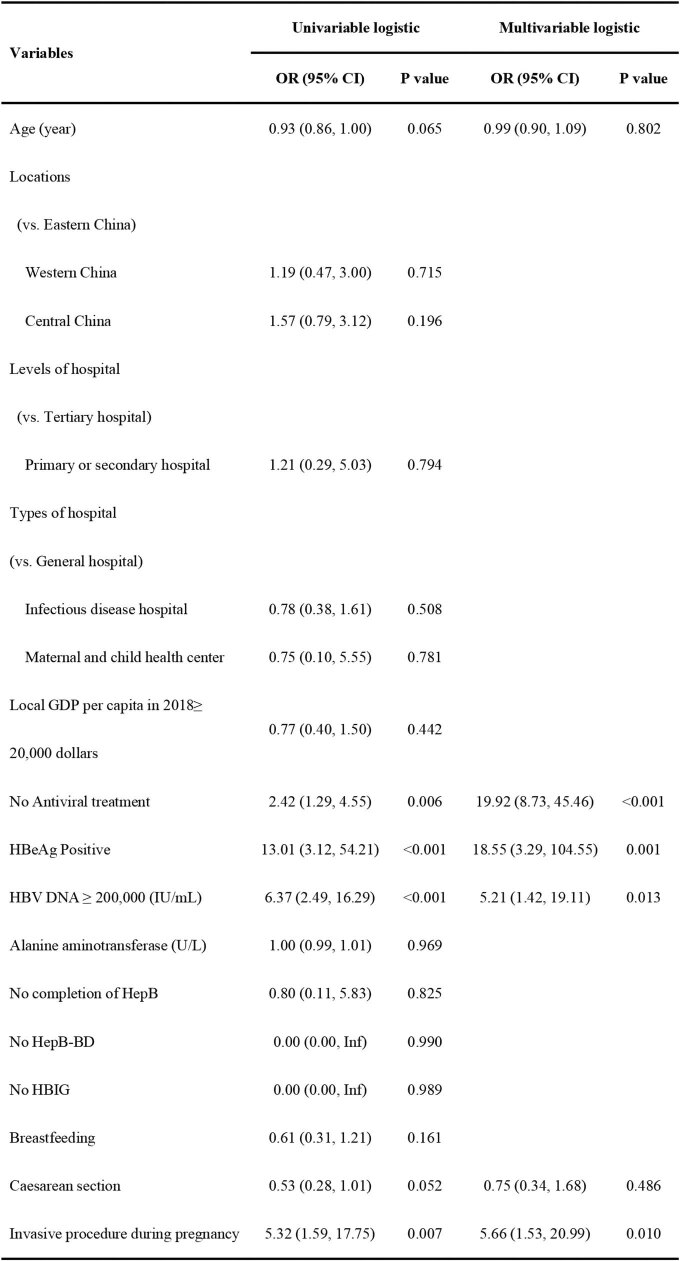
Logistic regression analysis on risk factors of mother-to-child transmission of hepatitis B virus in the implementation stage of SHIELD

We conducted logistic regression analysis on risk factors of mother-to-child transmission of hepatitis B virus in the implementation stage of SHIELD. Univariable logistic regression analysis was used to identify clinically relevant variables associated with MTCT in stage II. Collinearity diagnostics were conducted and variables (age (p = 0.065); no antiviral treatment (p = 0.006); HBeAg-positive (p < 0.001); HBV DNA ≥200,000 IU/mL (p < 0.001); cesarean section (p = 0.052); invasive procedure during pregnancy (p = 0.007)) with a p < 0.10 in the univariable logistic regression analysis were entered into the multivariable logistic regression model (no antiviral treatment (p < 0.001); HBeAg-positive (p = 0.001); HBV DNA ≥200,000 IU/mL (p = 0.013); invasive procedure during pregnancy (p = 0.010)). P values are two-sided. GDP, gross domestic product; HBeAg, hepatitis B e antigen; HepB-BD, hepatitis B vaccine birth dose; HBIG, hepatitis B immunoglobulin; HepB, hepatitis B vaccine.

## Data Availability

The clinical data are not publicly available as the program is still ongoing. The data will be available upon reasonable request for academic use only. If other investigators are interested in performing additional analysis, an application can be submitted to the corresponding author J.H., explaining the analyses planned. The request will be responded to within 1 month of receipt.
