# Supplementary material for: Real-world implementation of a multilevel interventions program to prevent mother-to-child transmission of HBV in China
Source: Nat Med. 2024 Jan 31;30(2):455–62. doi: 10.1038/s41591-023-02782-x (PMC10878969; doi:10.1038/s41591-023-02782-x)
Supplement: Supplementary file 1 — Supplementary SHIELD Study Investigators, Figs. 1–4, Note and research protocol. [file 41591_2023_2782_MOESM1_ESM.pdf]

nature medicine

Article

https://doi.org/10.1038/s41591-023-02782-x

Real-world implementation of a multilevel interventions program to prevent mother-to-child transmission of HBV in China

In the format provided by the authors and unedited

Supplementary information

Supplemental SHIELD Study Investigators

**Guangdong Province**			
Peifen Guo	Hongjun Li	Xiafei Fu	Guowei He
Xiaozhu Zhong	Chunxiao Wu	Yuanping Zhou	Ying Deng
Yingju Li	Yunfei Gao	Muhua Li	Yonghua Huang
Jinlin Hou	Peng Wang	Youfu Zhu	Qun Zhang
Zhefan Dai	Dongmei Hu	Yaping Wang	Suihua Feng
Yanying Huang	Mei Zhong	Yingying Huang	Chuangneng Lao
Manhua Zhong	Wenjun Zhang	Jie Peng	Yuanqiang Yang
Yaoyong Zhou	Liujuan Li	Ronglong Jiang	Linlin Lu
Zhanzhou Lin	Suiwen Wen	Xueru Yin	Shiyao Xian
Wenyu Mo	Hongying Zhu	Jian Sun	Xiaolu Liu
Canhui Xiao	Suran Huang	Huaiyu Chen	Weiping Chen
Bo Wan	Hongling Chen	Xiaorong Feng	Jia Wang
Jing Li	Jianjun He	Fuyuan Zhou	Xiaohua Li
Yanyan Yin	Lei Xiao	Weiqun Wen	Yan Tan
Qing Shan	Min Xu	Yongpeng Chen	MingcongZhao
Yurong Chen	Liling Li	Jinjun Chen	Changzheng Hu
Xiaoyan Li	Feifei Huang	Li Liu	GangHe
Huihua Liao	Yuyan Bai	Jinzhang Chen	Yueying Zhen
Weidong Luo	Yinong Ye	Xiaoyun Hu	Shaoqun Liang
Huiyuan Liu	Yuanling Xiao	Dingli Liu	Ming Luo
Biyan Liang	Xingliu Wu	Qiyuan Tang	Heming Wu
Hongshun Fan	Ming Chen	Dongying Xie	Xiaoqing Wang
Shu Yang	Li Liang	Zhenghua Ma	Zhijun Qu
Shilei Pan	Yu Quan	Xuemei Liang	Guimei Huang
Yingxia Liu	Yaping Lu	Honglian Bai	Daoyan Zhao
Suiqun Guo	Sujun Zhu	Caiqiue Cai	Hang Zhang
Youming Chen	Zhihong Liu	Jin Li	Jinyu Xia
Yingchun Li	Yuehua Chen	Jing Yuan	Xiaomou Peng
Yaotian Li	Pei Zhou	Xunhua Zhong	Fengyun You
Zheng Li	Keng Chen	Feijian Ao	Li Ding
Qixia Li	Qian Zhao	Simin Yao	Zhongsi Hong
Chenhong Wang	Yujie Li	Hong Yu	Chunna Li
Hong Yang	Yangyang Hu	Yanfeng Wang	Huili Chen
Zhihua Liu	CaixiaWang	Xueying Ruan	Mingxing Huang
Huixiu Zheng	Taojin Zeng	Lijuan Xiao	Jian Liu
Chuangguo Yang	Guiyu Gong	Haipeng Zhu	Xi Liu
Ling Song	Qingyu Li	Guixuan Chen	Zhaojuan Su
Qian Jiao	Jinfeng Ling	Jieqing Zhai	Jing Liu

Laiqin Peng	Mei Jiang	ZhongjunLi	Hongjun Sun
Yulan He	Yiping Luo	Wen Huang	Qiwen Yuan
Wenjian Li	Qi Zhang	Wanhua Wu	Niannian Chen
Songmei He	Yuanyuan Wang	Yuqing Li	Le Chen
Kewei Zhu	Yabing Guo	Lan Tang	Qi Li
Bei Zhong	Qinjun He	Chenhua Zhang	Ting Hong
Peng Zhang	Jinfang Zhou	Youming Chen	Miao Wang
Huafang Wang	Tianhuang Liu	Xiaohong Zhang	Yuanqiao Cheng
Ling You	Lihong Deng	Yongyu Mei	Wanjing Huang
Fan Yang	Zongyun He	Jing Lai	Yiping Zeng
Jun Wu	Zhancheng Yao	Jianguo Li	Qing Xu
Fangfang Zheng	Peishan Chen	Hongying Hou	Linli Sun
Jie Song	Xuan Zhou	Shuisheng Zhou	Yanru Lan
Zhizhong Deng	Xuan Li	Jianhui Fan	Youyuan Zhu
Youyou Wang	Zhifeng Chen	YuzhuYin	Shiwu Ma
Mo Chen	Xiaoxia Zheng	Zhenyan Han	Chong Zheng
Yangbin Guo	Ke Luo	Shibin Xie	Yuewen Guo
Sichun Yin	Congcong Li	Zhixin Zhao	Jianmin Luo
Yanzhong Peng	Zhenchang Wang	Hong Deng	Jiemei Liang
Guoxin Hu	Yue Su	Jianyun Zhu	Hongyu Zhang
Jing Wu	Lifa Zhang	Xuejun Li	Mingjing Lu
Lijia Chen	Rui Zhang	Lin Yang	Zhen Hu
Minghua Qi	Jun Xu	Yufeng Zhang	Lang Ming
Xindeng Tong	Xiaohua Chen	Yuehua Huang	Yong Huang
Ting Liu	Minfeng Liang	Chenghui Huang	Xiaoqiao Chen
Xiaohui Min	Hui Long	Aiqiang Zhou	Zhiqiao Zhang
Ruilie Chen	Ruixing Zhong	Chengyu Jiang	RongguoWang
Shaorui Lin	Qingyang Zhong	Cuiyu Feng	Yeqiong Zhang
Dinggang Zheng	Zongliang Li	Xinhua Li	Liang Peng
Xiangming Xiao	Peiqing Huang	Lifan Zhuang	Genglin Zhang
Lingjie Wu	Rufang Chen	Suqin Gan	Yanhui Ning
Haisheng Zhang	Haiming Yan	Xiuzhong Zeng	Zide Zhao
Genglong Guo	Wenjuan Tan	Shiliang Zhong	Jing Ma
Daqiao Zhou	Zhijian Yu	Zonghua Rao	Shaojun Zhu
Hui Gao	Duoyun Li	Xinzhi Zhang	Chunyan Lai
Yingjun Zheng	Jiong Yang	Jianhua Tian	Songmei He
Mei Qiu	Qin Yan	Zhemei Huang	Zhongbin Lin
GuilongZhuang	Yurong Wang	Hong Wang	Jinhong Yu
Ping Liu	Bing Bai	Lin Xiao	Yihong Chen
Yiqi Liang	Zengyou Liu	Xuegang Wang	Haidan Yang
XuliFu	Lin Zong	Qingmei Fu	Huizhong Kang
Guohang Li	Zhan Yang	Yao Liu	Xiaodan Zheng
Xi Yu	Peiyan Xu	Yingzi Long	Xiaoyi Fu

Jinying Mo	Boping Zhou	Yuejun Pan	Qingxian Cai
Jianyu Kuang	Guangdong Tong	Yuwei Tong	Xuanqiu He
Zhangjie Zhang	Fenghao Zeng	Xiaohong Ouyang	Hongbo Qin
Guoyu Tan	Lubiao Chen	Qiuli Xie	Li Zhuo
Yulin Zhan	Bingliang Lin	Guotao Lyu	Xiuhan Yang
Wei Zhao	Chaoshuang Lin	Lijiang Jiang	Jianxin Tan
Guoqiu Lin	Jing Liu	Chunrong Huang	Yongyin Li
Yiping Luo	JieZheng	Junchao Qiu	Xin Wei
Meijie Shi	Ping He	Chengwei Zhou	Jie Dong
Jiayi Xie	Fang Guo	Keli Yang	Qing Yang
Pu Wang	Huiqian Zeng	Damei Zhou	Yanhong Chen
Danchun Cai	Wanqing Ji	Wenting Zeng	Qian Li
Jia Shi	Yuewei Jiang	Zhihui He	Dan Chai
Huaichang Liu	Shufang Yao	Ling Yang	Xiaohui Liao
Miaogen Li	Hongbo Gao	Jing Shi	Yu Li
Xinghai Zhao	Miaoxin Lu	Hao Zhang	Hong Sun
Haibo Lou	Yanqiong Liu	Maomao He	HaiqunXiao
Yumei Chen	Zhaodi Huang	Ruqing Zhao	Zhiyi He
Huijie Guo	Wei Wang	Qiwei Luo	Yaping Gan
Xihua Fu	Meiting Huang	Yanwen Xu	Jianxin Liang
Guobin Zhao	Jinna Li	Yanqiu Li	Huanlian Wen
Guocheng Liu	Wanzhen Chen	Qiling Huang	Xushuo Xie
Yiping Luo	Xiaoxia Huang	Jing Wu	He Zhang
Weijian Li	Wanmei Hong	Aiqin Yuan	Yingyan Qu
Zhen Guo	Yujie Ren	Cuixia Zhong	Xiaohan Huang
Yan Peng	Meilan Guo	Jinwei Guo	Zengwei Liang
Chunling Hu	Meiling Yin	Jing Zhou	Weiyuan Liu
Limei Fan	Yuhua Zhang	Yuqian Ren	Weiyuan Liu
LikunXu	Qianwen Yang	Peng Hu	Lihua Wu
Liqun Li	Lingfang Long	Zhixiang Zhang	Jianhong Xia
Changqing Lin	Jundong Li	Jianen Yang	Liqi Su
Liuyu He	Huiqing Gao	Baomin Yin	Shi Ouyang
Yan Zhang	Qing Zeng	Jihong Li	Fangming Liu
Xiaofei Lyu	Qianhua Zhang	Fengmei Jin	Shuangming Cai
Huihua Liu	Dandan Hua	Xiaohong Yang	Tingting Peng
Yiting Liu	Xia Cai	Changli Duan	Haijian Fan
Wenhui Ye	Meiling Zeng	Li Liu	Guanglin Li
Yunzhong Guo	Pin He	Yingting Chen	Lili Yang
Si Chen	Hongxia Guo	Chunyan Chen	Xuan Zhong
Guang Shao	Wenting Mo	Xiaoxia Li	Yu Liu
Jinbin Lai	Xiujuan Zhang	LinfengHong	Lina Wang
Xicai Wu	Fang Wang	Xiaoling Liu	Jinfeng Liu
Louyu Chen	Linjun Zhou	Hongxia Zhang	Qiong Liang

Huachao Mai	DanWu	Lili An	Shan Huang
Wenni Zhang	Haifei Luo	Weiying Dong	Danfeng Yu
Huanshun Xiao	Guoxin Liufu	Lihong Lin	
**Anhui Province**			
Qiuping Dong	Jiabin Li	Yunlan Chen	Duanduan Zhou
Qian Duanmu	Fei Su	Yun Wang	Hui Zhang
Hui Wang	Qian Su	Yeying Ding	Nana Ji
Linghua Tang	Heng Sun	Li Yu	Xiaosu Zhang
Fenghua Wang	Qiulin Sun	Chunling Mao	Yuchen Pan
Xiujuan Li	Lingling Xia	Wei Zhang	Xingxing Jiang
Feng He	Qinxiu Xie	Panpan Zhang	Huizhi Huang
Weishun Hou	Xihai Xu	Hui Xue	Xuelian Zhang
Huafa Yin	Ying Ye	Jinhua Zhang	Lebin Wang
Zhongjing Xia	Zhenhua Zhang	Yiqun Huang	Chuanfu Wang
Li Ma	Qingling Zhang	Xiuyan Chen	Ling Wang
Zhongsong Zhou	Ce Chen	Caimei Gu	Fengsheng Jia
Jun Cheng	Xuefeng Dai	Zhi Li	Chunlin Jiang
Xiaoping Jiang	Jiaqi Liu	Shuxia Cao	Jing Wang
Hongbin Li	Zhaogang Cheng		
**Beijing**			
Qian Bian	Hua Zhang	Lili Liu	Qinqin Wang
Ming Wang	Yunxia Zhu	Jinfeng Chu	Wenjuan Pu
Bo Li	Huili Liang	Xiufang Yu	Mei Shi
Xin Liu	Lai Wei	Mingliang Dong	Shuai Wang
Xiang Gao	Bing Zhu	Dandan Li	Juqiang Han
Ruihua Tian	Yinghui Yin	Wei Zhao	Jinhua Xiong
Mei Wang	Xia Liu	Haiwei Sun	Yongqiang Ren
Lingzhi Chang	Xiaohui Liu	Jingfang Ren	Wei Wen
Chong Zhang	Jinhua Wang	Li Li	Yiming Zhou
**Fujian Province**			
Qianguo Mao	Chuncheng Wu	Jing Dong	Xiaowen Chen
Jinmo Tang	Yue Chen	Jing Chen	Yixian Shi
Jiaen Yang	Manying Zhang	Youbing Li	Naling Kang
Chong Gu	Jiaji Jiang	Dawu Zeng	Shengtong Weng
Lijian Huang	Yueyong Zhu	Su Lin	Lei Zheng
Ying Zheng	Qi Zheng	Yihong Chai	Liqing Zheng
Huiqing Liang	Jia You	Jiumei Zhang	Lyufeng Yao
Xianqiong Gong	Min Wang		
**Gansu Province**			
Fang Wang	Xinren Zhou	Jia Wei	Yan Wang
Shuxian Feng	Shenghao Yun	Nina Wang	Lin Pan
Junfeng Kou	Ruili Chen	Qian Chen	Wenfan Li
**The Guangxi Zhuang Autonomous Region**			

Qian Guo	Shengping Yin	Chunlian Meng	Yuting Bao
Jianghong Chen	Minghao Qiu	Renguo Lei	Ren Chen
Cunli Nong	Ping Tan	Fen Li	Dan Xu
Xiling Mo	Huihui Wu	Shuzhen Wei	Jingjiao Lei
Yufang Luo	Qiufang Wei	Dongyun He	Jinqiu Huang
Tingting Li	Xuehan Peng	Hua Meng	Yelin Wei
Jing Guan	Yingwei Li	Qin Hu	Cuimin Wang
Jun Meng	Lianhua Pan	Qinghua Lu	
**Guizhou Province**			
Qianjun Ren	Xiaoqiong Gou	Quan Zhang	Xiaocui Yang
Lingling Ma	Yang Li	Tianyong Luo	Jiuqian Li
Yanbing Xiao	Liqian Yang	Yayun Wu	Xiulan Zhang
Li Zhang	Yun Long	Shuang Lu	Qixiang Li
Ya Han	Yuanhong Liang	Mao Mu	Li Zhang
Lixia Wu	Mingliang Cheng	Baofang Zhang	Xinhua Luo
Dingying Yang	JunWu	Qin Liu	Tianzhao Liu
Jisha Du	Jing Yang	Kaisheng Deng	Mingjuan Zhu
Benshan Peng			
**Hainan Province**			
Biao Wu	Li Shi	Ping Qiu	Yanteng Zhou
Furong Xiao	Hui Gao	Xiaozhen Xu	Jian Fu
Feng Lin	Guanghua Pan	Shiming Zhou	Xiuchun Zhang
Suoxian Chen	Wei Shen	Duyun Cai	Baiyu Pan
Tao Wu	Ying Wang	Yuanxue He	Jiao Wang
Xiaoli Fu			
**Hebei Province**			
Yang Yang	Jianxia Li	Bo Li	Ying Qin
Huanwei Zheng	Caiyan Zhao	Jian Wang	Changfen Wu
Xiuli Chen	Fang Liu	Xiaojun Liu	Wei Wang
Baoshen Zhu	Hongzhu Yin	Jingru Ma	Dongxiang Han
Suwen Li	Yadong Wang	Zhongfu Mo	Jinyu Gu
Jianping Xu	Luyuan Ma	Xiaolei Cao	Siyu Li
Hongxia Tian	Qian Zhao	Chunyan Yu	Cuili Yang
Lijuan Sun	Wei Wang	Jing Liu	Yuchan Zhao
**Henan Province**			
Heping Zhao	Hui Yin	Liujiang Bai	Peng Wang
Jia Shang	Huibin Ning	Lingju Wang	Shuai Guo
Yu Wang	Ling Jiang	Zhijie Mu	Xiandong Liang
Yi Kang	Guoqiang Zhang	Pan Zhang	Wenliang Zhou
Hewen Wu	Jujun Hai	Yuanliang Huang	Ying Feng
Chongshan Mao	Fang Wang	Zhenhua Wang	Guotao Li
Junfeng Wei	Fengqi Han	Shixi Zhang	Chunyan Zhou
Junping Liu	Yujie Tan	Peipei Wang	Lihua Zhang

Li Wang	Yalan Sun	Juan Li	Liantao Zhang
Wei Li	Yumeng Zhu	Zan Shi	HuanrongHou
Zhen Peng	Danyan Zhu	Kai Li	Fengxian Yu
Jinhuan Qin			
**Heilongjiang Province**			
Yujie Zhao	Jingnan Shao	Ming Mao	Long Zhao
Liying Zhu	Hao Zhang	Xuguang Zhu	Xiwei Gong
Lihua Zhong	Jie Chi	Yanhua Xiao	Hongyan Zhang
Yun Wang	Xuwei Qin	Haiyan Wang	Yanlei Zhang
Lei Yu	Ying Shen	Zhichao Shao	Jingting Bi
Xiaohua Yang	Changqing Li	Juan Du	Wenyan Gong
Baoling Lu	Yanqiao Shi	Li Yin	Hongxiu Zhao
Yu Cheng	Yuan Tian	Yanli Dong	Dan Ma
Hong Yao	Yanbo Wang	Lei Pang	Nannan Zhao
Jian Fan	Guojun Zhang	Wanming Zhu	Dandan Dong
Yuanyuan Wang	Zhiying Fan	Ying Liu	Jing Wang
Guimei Liu	Ying Dai	Mingjing Wang	Yuxiu Song
Shu Guo	Li Cao	Qiu Wang	Li Gao
Wei Zhang	Tianwei Liu	Xiuli Jia	Yanwei Xu
Jinping Zhang	Yue Shi	Lijing Wang	Zenghui Li
Tao Huang	Jing Han	Hongmei Cao	Wei Yu
Binghua Yang	Jing Liu	Wei Zhang	Jieqiong Hou
Ran Liu	XinZhang	Tiansheng Cao	Xiqiu Zeng
Sen Liu	Shanshan Yang	Yanxia Zhang	Yumei Zhang
Huan Qi	Shimin Wang	Ran Liu	Huaixiu Wu
Dan Zhang	Lihui Feng	Wei Hu	Bolin E
Jingwen Zhang	Wenhan Yang	Yanjuan Cheng	Qiu Fu
Jinmei Feng	Weiling Zhang	Yuhua Cui	Mingzhu Wang
Yan Yue	Liwei Ji	Guoli Yan	Wei Wu
Weiwei Li	Yunsong Zhang	Li Zhou	Wanming Zhu
Jinwei Li	Liping Yu	Lili Wang	Yu Zhang
Jiqing Li	Dongying Fu	Haiyan Liu	Chao Wei
Wei Guan	Mingjie Sun	Shanshan Chen	Litao Liu
Hongyan Gai	Qiulin Wang		
**Hubei Province**			
Shaonan Yan	Zhiyong Zhang	Wanjiang Zeng	Min Chen
Bin Deng	Xiaobei Chen	Zuobing Wang	Jing Zhang
Xiaojing Jiang	Fan Yang	Cuifang Zhang	Wenmin Fang
Futao Zhao	Shiyun Guan	Jianfeng Yuan	Liming Li
Ying Liu	Shundong Huang	Fan Zhou	Qian Yang
Junli He	Ling Feng	Ping Zhou	Deng Pan
Le Song	Dean Tian	Jiangang Wang	Liqiong Huang
Guiyue Shen	Deying Tian	Zefu Nie	Yuye Shen

Xin Wang	Zhengang Zhang	Jun Yu	Jun Zhu
Hanyun Yang	Wangxian Tang		
**Hunan Province**			
Jun Quan	Lihua Hu	Feng Yi	Ling Wang
Yan Huang	Xiangzhen Long	Chihua Liu	Yukun Huang
Jianping Xie	Xiaomin Wang	Yunhua Zhu	Congzhi Li
Yuanyuan Wang	Yulan Jiang	Xiaofen Li	Fei Liu
**Jilin Province**			
Nan Zhao	Shuqin Zhang	Yulin Chu	Ying Song
Weibing Tong	Jing Jiang	Wei Liu	Wei Li
Yuqing Yan	Chong Wang	Xi Chen	HuiChen
Ru Guo	Lishu Zhang	Yuhuan Wu	Yuting Wang
Guiqing Chen	Haiying Sun	Qiulian Li	Bo Sun
Chang Shu	Ming Wang	Quan Sun	Bo Wang
Hongmei Xu	Yanling Li	Min He	Yuxi Ma
**Jiangsu Province**			
Guorong Han	Li Xiao	Lihua Ye	Xiaoyun Zhang
Hongxiu Jiang	Xiuzhen Yang	Juanjuan Fu	Liping Wang
Sugui Cheng	Yang Li	Dongmei Wang	Qin Ding
Zhengru Zhang	Bian Wang	Yongyan Tang	Guangde Yang
Xiuhua Sun	Wei Wang	Chancong Gong	Li Li
Mei Luo	Libing Han	Genju Wang	Yan Liu
Mengmeng Du	Aiwen Geng	Xin Yue	Chunyang Li
Lili Yang	Meilong Shen	Xiaoxia Tang	Wei Yao
Yinling Zhao	Xinhua Bu	Yu Zhang	Xiaohong Guo
Junhua Wu	HezhuWang	Xiaofang Jiang	Li Gong
Li Jiang	Aihua Huang	Xueping Li	Yi Ding
Shaojun Wu	Haiyan Jiang	Huibing Sun	Shufen Bai
Xiaoxiang Wang	Yiqun Wu	Shasha Luo	Guifang Gu
Cen Xu	Shanshan Shao	Aihua Kong	Min Su
Jing Wei	Lihua Huang	Xiaomei Ding	Ji Li
Hongzhan Sun	Bo Zhang	Chuanwu Zhu	Lin Ye
Lin Wang	Shangzhi Yao	Jie Yao	Chunyan Ge
Hongfang Ju	Zhong Hua	Deming Ma	Yan Chen
Hua Qian	Tong Sun	Yu Zhang	Ren Qiang
Chunyan Ye	Yunchuan Pu	Lina Zhang	Aimin Cui
Defang Zhai	Zheng Wang	Xiaoying Yao	Jinxia Xu
Xueming Zhang	Qien Yang	Rong Zhang	Sue Jiang
Jianhong Pei	Xinguo Wang	Xuebing Yan	Wen Xu
Zheng Wei	Xiaoxia Tang	Xiucheng Pan	Yuxi Ma
Jianhua Jiang	Jianchun Xian	Ming Chen	Fangzheng Han
Hui Zhang	Zhongqin Wang		
**Jiangxi Province**			

Guojun Shen	Yun Luo	Min Kong	Peihua Yang
Ming Li	Ningning Wang	Guilian Zhong	Jingke Zeng
Lu Shao	Min Yang	Li Rui	Lijuan Long
Yilei Tao	Xiaolin Zhang	Xin Huang	Yuling Lan
Yushan Lu	Shiqiong Zhou	Yinbai Fan	Xia Wang
Lifeng Cao	Liying Zhou	Xiaoxiong Hu	Wangui Zhang
Yun Hu	Yuan Fang	Meiling Huang	Dixiu Liu
Jing Zhang	Tao Yue	Manlei Jiang	Guanlin Zhou
Yao Wen	Fenglin Kong	Fei Xu	Xiaolan Wang
Shengping Fang			
**Liaoning Province**			
Yang Ding	Danyang Liu	Shu Sun	Lilan Shi
Xiaoguang Dou	Qiong Liu	Fang Zhan	Jingyan Wang
Qiuju Sheng	Qingwei Gao	Yan Wang	Lin Zhang
Chong Qiao	Xin Liu	Lin Bi	Han Bai
Jun Wei	Yanshan Liu	Baijun Li	Jun Ran
Zhuo Feng	Fengmin Xu	Fen Huang	Yong Wang
Bin Ning	Shijie Lyu	Xuelian Wang	Ping An
**Inner Mongolia**			
Zhongsheng Liu	Yan Zhang	Jinlian Zhou	Huiting Wang
Shuyi Suo	Guirui Bai	Meiying Shi	Haiyan He
Huanhuan Liu	Hong Xing	Guiying Nie	Na Ta
Min Li	Lulin Wang	Xinyue Wang	Huiyun Xu
Feiyun Bai	Hong Fan		
**The Ningxia Hui Autonomous Region**			
Xiangchun Ding			
**Qinghai Province**			
Hongmei Zu	Shengrong Zhang	Yu Zhang	Xiaoyan Zhang
Qinghua Lu	Junning Peng	Jiaying Yan	Hongmei Duo
Haifang Cao	Hude Wang		
**Shandong Province**			
Feng Gao	Suping Zhang	Xia Cui	Lu Guo
Qingyan Li	Hongxia Wang	Jianguo Yuan	Ru Chen
Xiaoge Yang	Ronghua Liu	Hongkui Zhao	Wenmei Chen
Xia Li	Ruxiu Xu	Qingfeng Shi	Lin Zhang
Xuejun Liu	Yanyun Wang	Zhenzhou Xu	Xiuping Yan
Lin Li	Tian Shao	Mei Zhang	Fenghua Liu
Xiumei Chen	Yali Cheng	Xin Wei	Xiaonuo Gao
Hong Wei	Yujun Liu	Wei Lu	Xia Li
Ruili Mou	Yunguang Li	Zhanjie Niu	Li Lin
Min Song	Qi Chen	Xue Li	Xin Geng
Xiaoping Li	Qingping Gao	Huanrong Feng	Yan Wang
Cuilan Wang	Jing Du	Linlin Sun	Qiaozhen Chen

Dong Hao	Sikui Wang	Shuxia Ge	Hui Lyu
Tingting Zheng	Shengru Zhang	Linuo Peng	Jilan Yu
Caixia Chen	Yuanzheng Gao	Xiaobing Wang	Jing Du
Guoguie Guo	Qingfang Li	Tong Yuan	Jie Li
Jian Li	Shan Guan	Rendong Wei	Huibin Zhu
Yuan Wu	Wei Chen	Jingjing Guo	Qinge Gao
Cuizhi Li	Peng Ning		
**Shanxi Province**			
Xihong Wang	Bo Wang	Jianmin Rong	Cuipeng Feng
Xiang Ma	Rui Li	Haiyan Zhu	Caihong Wu
Xiaoli Liang	Yiqun Qu	Xining Wang	
**Shaanxi Province**			
Tianyan Chen	Jiuping Wang	Yanjun Li	Jiejing Xin
Yingren Zhao	Ruifeng Tian	Tai Wang	Chunxia Li
Jinfeng Liu	Yu Liu	Jihong Feng	Bing Dong
Yuan Yang	Wei Zhang	Na Liu	Yutao Liu
Yingli He	Yage Zhu	Peidong Zhao	Chunyan Li
Taotao Yan	Feng Ding	Junxiao Qu	Pingping Zhang
Zhen Tian	Dan Liu	Yafang Zhang	Yuan Zhao
Dandan Guo	Guanghua Xu	Huaiqiang Pan	Yidan Zhang
Rongfang Xu	Xiaohong Gao	Chengfu Wang	Le Yao
Zhigang Liu			
**Shanghai**			
Lihui Jiang	Jun Zhao	Rui Guan	Junyao Lu
Li Yan	Chengzhong Li	Jinfeng Zeng	Lei Yan
Zhimin Han	Xuesong Liang	Peiru Jiang	Yupan Bai
Min Liu	Jianya Xue	Hongmei Deng	Yuanyuan Zhou
Xiaohong Zhang	Zhihui Chen	Jinghua Liu	Yaoyue Kang
Jielian Yang	Ruiying Zheng	Jie Xu	Yue Li
Yangqiu Chen	Jixiu Chen	Qin Fan	Hongjuan Chai
Minmin Sheng	Wei Yin	Xiaoling Yuan	Yunhui Zhuo
Jie Yang	Yuhuan Liu	Shengzhen Hong	Bei Luo
Weiwei Sun	Yu Chen	Donglin Yin	Chuanlou Xu
Jiayan Gu	Lin Zhou	Xue Yang	Lihong Qu
Fengdi Zhang	Yingqiu Shen		
**Sichuan Province**			
Min Zhou	Enqiang Chen	Xiaomei Zhong	Xiaoxia Geng
Kejing He	Lingyao Du	Yi Zeng	Xuebing Chen
Huachun Yin	Libo Yan	Li Yin	Jiahong Yang
Juan Li	Hong Li	Qijun Cheng	Feifei Liu
Zhaohui Zhu	Juan Tang	Limin Zhou	Wanrong Luo
Mingxiang Wu	Chunfang You	Tingting Luo	Bibo Wu
Yufen Li	Jing Tang	Jianmei Lin	Fuli Shu

Haixia Huang	Wei Deng	Xingxiang Yang	Shushu Liu
Lang Bai	Yijun Liu	Renguo Yang	Han Zhuang
Hong Tang	Jianli Xu	Rengang Huang	Rong Hu
Shuqiang Wang	Xia Zhu		
**Tianjin**			
Hai Li	Xin Guo	Jinyu Hao	Huiying Yang
Shumin Ning	Jing Chen	Jing Hao	Yanli Shi
Yurong Zhang			
**Tibet**			
Li Shi	Deji Ciren	Qiongda Cidan	Bazhen Ciwang
Sang Ba	Panduo Dawa	Panduo Laba	Panduo Laba
Qingping Wen	Quwang Danzeng	Wenfan Luo	Quanyan Zhu
Lamu Mima	Lamu Bianba	Ciren Tudan	Daoping Han
Zhenzhen Wu			
**The Xinjiang Uyghur Autonomous Region**			
Qin Xu	Hongfeng Wang	Feng Guo	Zhuanguo Wang
Xiaozhong Wang	Huxibaiheti	Xiaobo Wang	Dan Han
Ka Ni	Yan Ma	Xiaofang Zhuang	Jie Zhang
Yonghong Yue	YanWang	Qiang Fu	
**Yunnan Province**			
Jing You	Hongli Zhang	Lu Zhang	Yilan Xia
Jinghua Fan	Junxin Zhang	Jiawei Geng	Ling Zhu
Guowei Li	Yihui Chen	Wei Yue	Xiaoqing Wang
Wu Li	Chunmei Chao	Yulong Wang	Xiao Liang
Hong Dai	Yanmei Zhang	Bing Bu	Xiuying Ma
Weibo Yang	Xianli Li	Liping Huang	Ruyi Zhang
Ying Niu	Ju Zhou		
**Zhejiang Province**			
Suying Zhang	Wanfeng Hu	Lingyan Shen	Chuantong Lu
Shourong Liu	Hongliang Ou	Hua Xuan	Yingming Fei
Xin Luo	Lingyan Fan	Xu Wang	Furong Liu
Zhe Yu	Xijie Lai	Danfeng Sun	Fuyan Sun
Chun Zhao	Chen Wang	Xiao Yu	Tao Xiong
Xiankai Wang	Xiaofeng Guo	Min Deng	Hongping Xuan
Pei Hu	Chengjing Tao	Jianming Wu	Jie Jin
Yaoren Hu	Li Tian	Fanchun Fu	Wenbao Huang
Airong Hu	Dongfang Ni	Xuzhen Lu	Xiaoxian Jiang
Xiunong Jiang	Xinsheng Xie	Qianqian Zhou	Lifei Yu
Wen Zhang	Xiong Sheng	Jieping Li	Jinfeng Shi
Jiong Wan	Yunqing Chen	Biao Zhu	Yi Jiang
Wenying Jiang	Hong Wang	Lin Qiu	Xiaoxiao Liu
Lingyun Zhang	Xiaofu Yang	Jie Wang	Chenwei Pan
Guoxian Zhu	Xiaoxia Bai	Yanting Bao	Aiqun Ren

Min Wu	Ran Ding	Suzhao Pan	Xiaobo Ying
Xiang Zhou	Deng Huang	Lifang Guo	Ligang Xu
Lidan Zhang	Wangwang Xu	Jiguang Ding	Yi Lin
Jing Zhu	Bingqi Ye	Qingwei Du	Gongying Chen
Huiqin Li	Tingting Pan	Danmin Wang	Yafen Qiu
Caixia Xia	Liqin Yu	Hongying Pan	Danhong Yang
**Chongqing**			
Wanju Yang	Fengying Wang	Xueyan Wang	Yi Wu
Wenling Cai	Qing Mao	Jie Xia	Wei Sun
Hua Hu	Ting Xie	Junnan Li	Chuwen Li
Qinghua Zhang	Xiaohong Wang	Lan Tao	

Supplementary Figure 1. The comparison between the SHIELD program and iPMTCT program (A, B) and the framework of the SHIELD program (C).

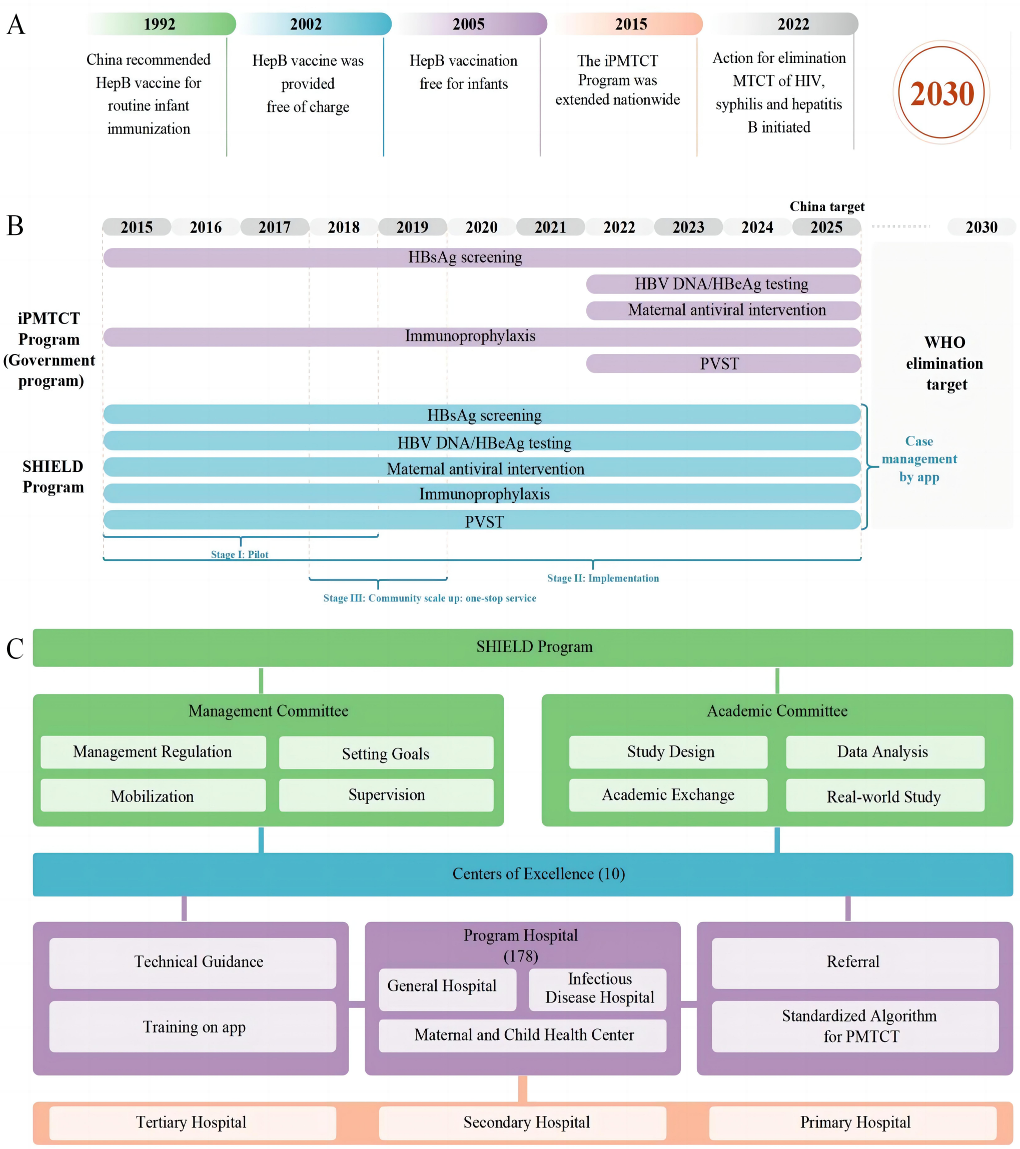

A, Major government policies to prevent HBV MTCT in China. **B,** The SHIELD program is a vital complement to the existing government iPMTCT program. In real-world practice, the SHIELD program integrated HBV DNA viral load, HBeAg, maternal antiviral intervention and HBV-exposed infant PVST into the management algorithm for preventing HBV MTCT since 2015, and the iPMTCT program has

integrated these nationwide since 2022. Besides that, the SHIELD program developed an app to facilitate patient management. Furthermore, the SHIELD program explored a one-stop service for managing PMTCT of HBV in stage III, which has the great potential to improve treatment uptake and compliance. Currently, the novel one-stop service has been adopted by the government. **C,** The SHIELD program established a Management Committee responsible for management regulation, setting goals, mobilization and supervision, and an Academic Committee responsible for study design, data analysis, academic exchange and real-world study. The SHIELD program consists of 10 centers of excellence (COEs) and 178 member hospitals nationwide, including general hospitals, infectious disease hospitals and maternal and child health center, tertiary, secondary and primary hospitals. Ten hospitals were chosen as COEs due to their experiences and skills in the field of PMTCT. The COEs were responsible for technical guidance, referral, training the medical staff from nearby member hospitals on algorithm and app and ensuring that the management algorithm was correctly followed. hepatitis B vaccine, HepB; iPMTCT Program, the Chinese National Integrated Prevention of Mother-to-Child Transmission of HIV, Syphilis and Hepatitis B Program; HBV, hepatitis B virus; MTCT, mother-to-child transmission; HBsAg, hepatitis B surface antigen; PVST, postvaccination serological testing; HIV, human immunodeficiency virus; WHO, World Health Organization.

Supplementary Figure 2. Service for preventing mother-to-child transmission of hepatitis B virus in the scale up stage of SHIELD.

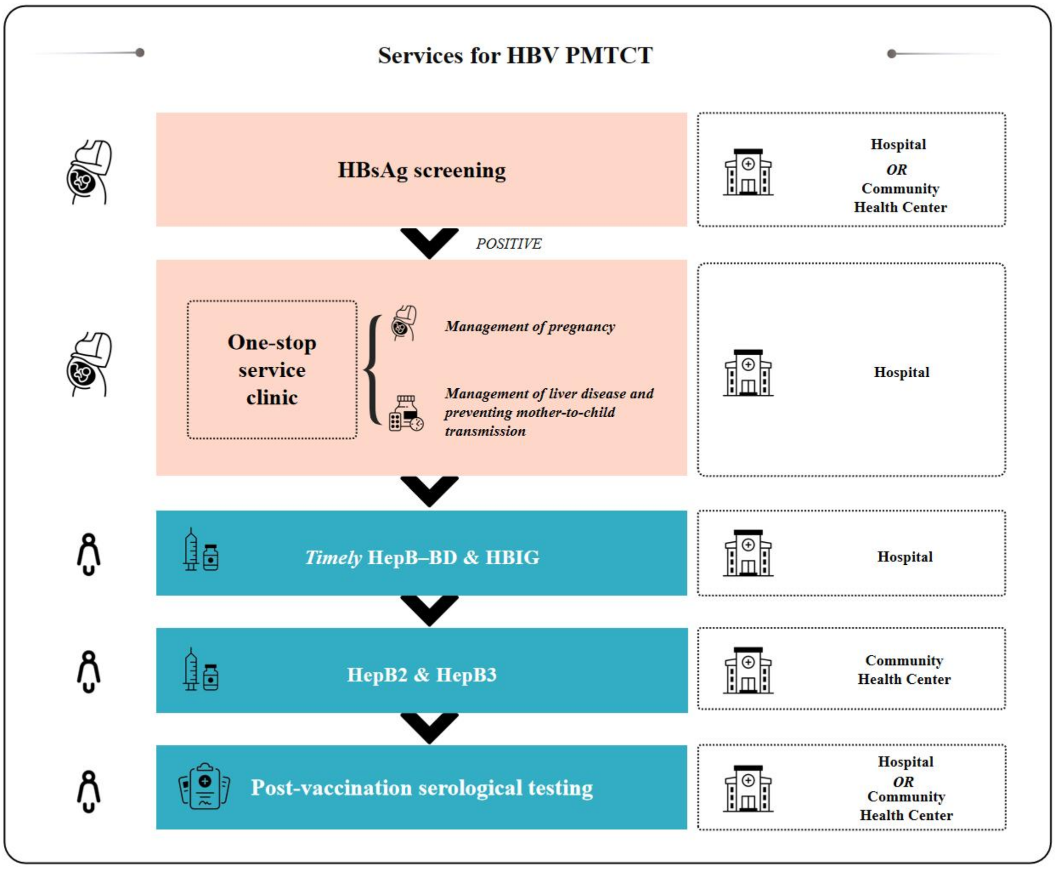

In the scale up stage of SHIELD program, HBsAg screening was provided in hospital or community health center. One-stop services were especially established in all Bao'an district hospitals to implement the intervention package for all pregnant women with HBV infection. Infants received HepB-BDs and HBIG in hospitals, and received the other two HBV vaccine doses in community health centers. Postvaccination serological testing was performed in hospital or community health center. HBsAg, hepatitis B surface antigen; HepB-BD, hepatitis B vaccine birth dose; HBIG, hepatitis B immunoglobulin; HepB2, hepatitis B vaccine second dose; HepB3, hepatitis B vaccine third dose.

Supplementary Figure 3. Algorithm for preventing mother-to-child transmission of hepatitis B virus.

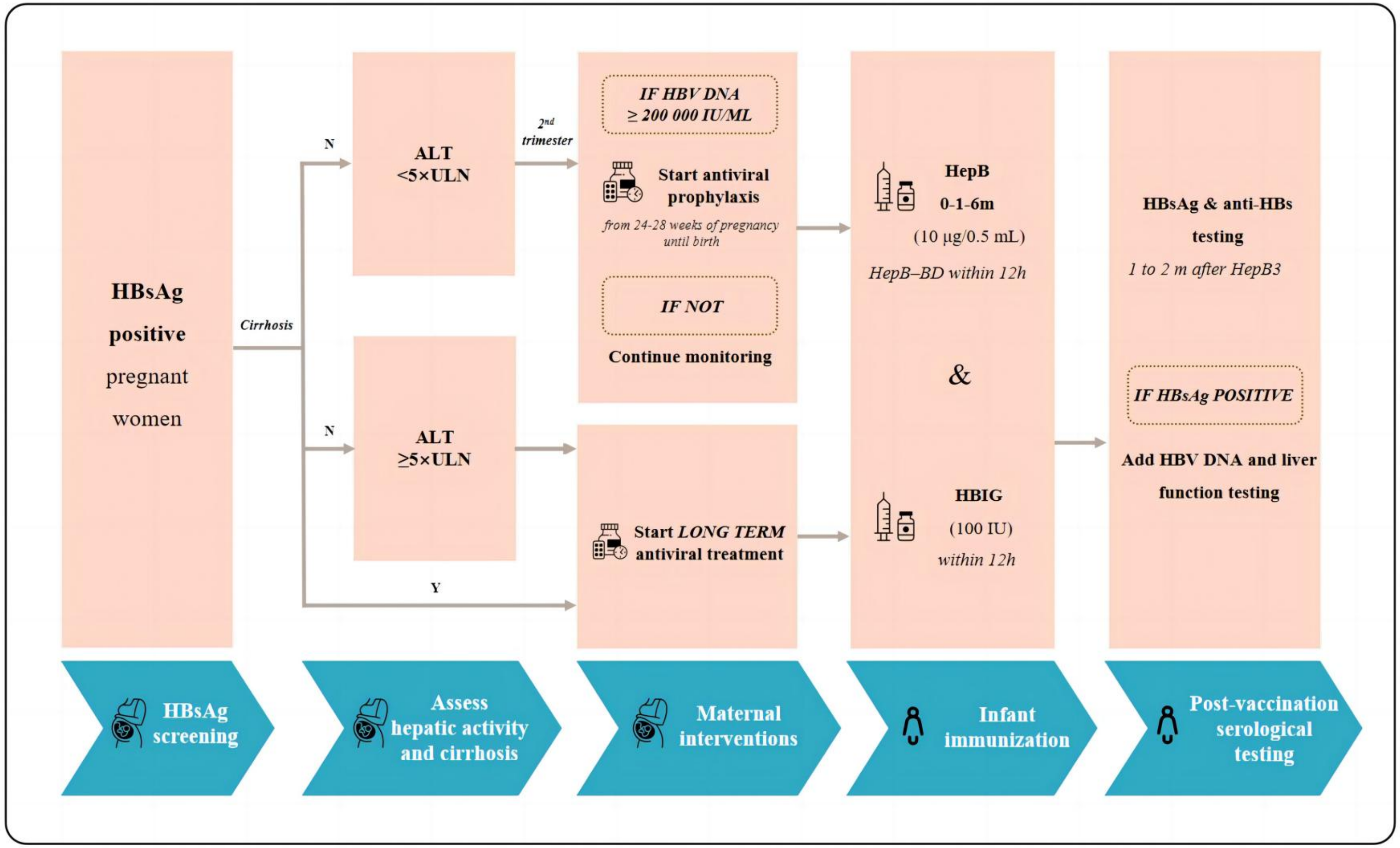

Antenatal HBsAg testing is universally and routinely offered to all pregnant women based on the iPMTCT Programme in China. Pregnant women with positive antenatal HBsAg tests are linked to appropriate clinical care services for managing chronic HBV infection. The HBV infection status of the enrolled pregnant women was assessed at baseline. Pregnant women with evidence of cirrhosis or significant hepatic activity with an alanine aminotransferase (ALT) level ≥ 5 times the upper limit of normal (ULN) were started on long-term antiviral treatment. Pregnant women with no evidence of cirrhosis or significant hepatic activity were monitored at least every four weeks during pregnancy and were administered antiviral treatment if indicated. Pregnant women who did not require antiviral treatment but had a high HBV DNA viral load (the eligibility criterion was ≥200,000 IU/mL) were considered at increased risk of HBV MTCT. Maternal antiviral prophylaxis, using tenofovir disoproxil fumarate (TDF) or telbivudine (LdT), was initiated at 24-28 weeks gestation and continued until delivery. Infants received the HepB-BDs (10 μg/0.5 mL) and HBIG (100 IU) as soon as possible after delivery (within 12 hours). The other two HBV vaccine doses (10 μg/0.5 mL) were scheduled at one and six months of age following the national Chinese vaccination schedule. PVST was performed after the completion of the HBV vaccine series and at least one month after the last HBV vaccine dose (at ages 7-12 months). HBsAg, hepatitis B surface antigen; ALT, alanine aminotransferase; ULN, upper limit of normal; HBV, hepatitis B virus; HepB, hepatitis B vaccine; HepB-BD, hepatitis B vaccine birth dose; HBIG, hepatitis B immunoglobulin; HepB3, hepatitis B vaccine third dose.

Supplementary Figure 4. The interface of SHIELD application.

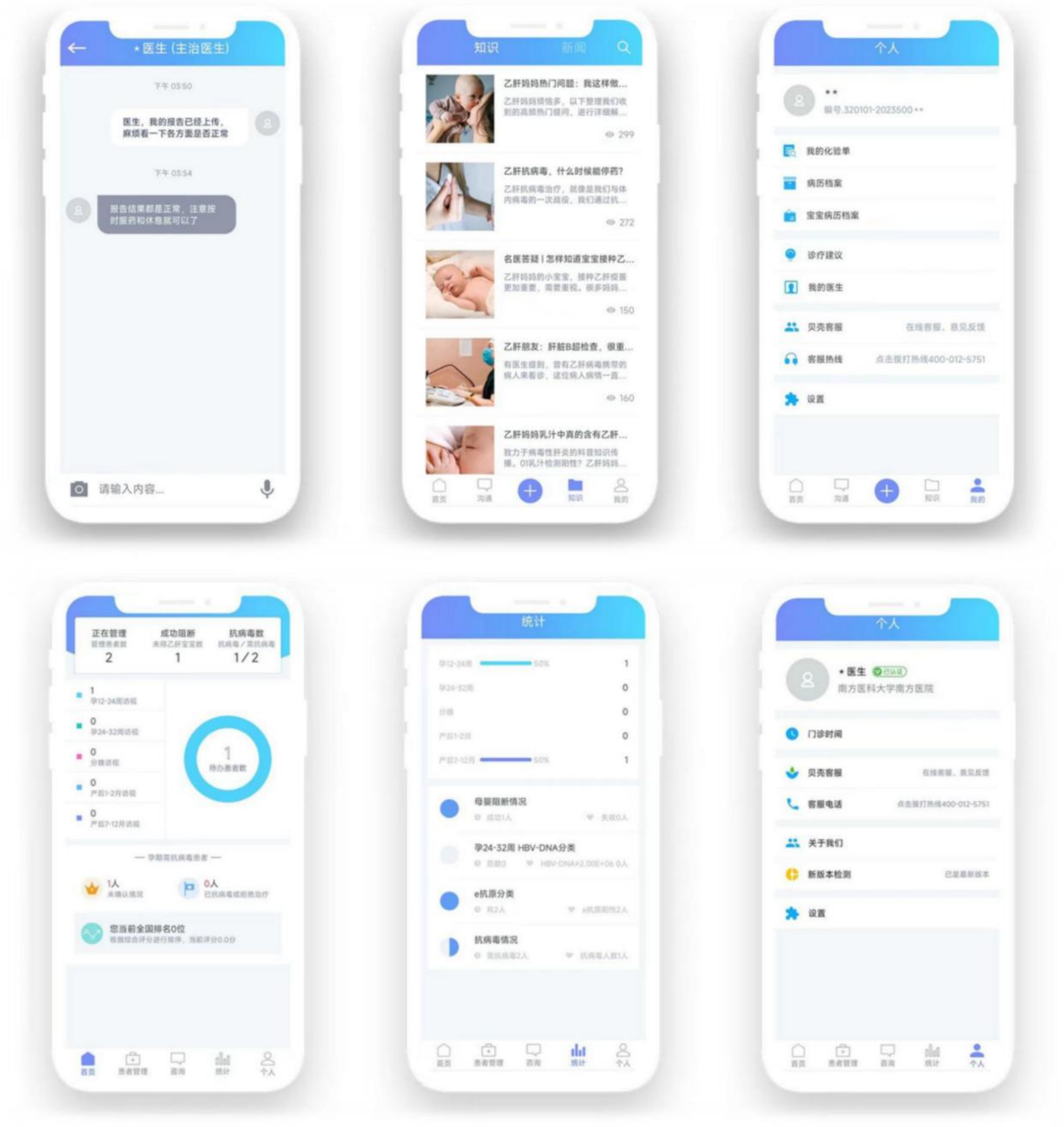

A mobile health application called the "SHIELD app" was developed. Participants could consult with their doctors for free via the SHIELD app during follow-up. The participants captured all laboratory test reports as pictures and uploaded them into the SHIELD app.

Supplementary document 1

Management algorithm for preventing mother-to-child transmission of hepatitis B virus

The management algorithm for the care of HBsAg positive pregnant women and their infants[1], is as follows:

1.Testing and diagnosis

Following current national recommendations of National Health and Family Planning Committee (NHFPC), all pregnant women should be universally screened for hepatitis B, syphilis, and HIV at the first prenatal examination and/or at the earliest time during antenatal care.[2] HBV serologic markers include hepatitis B surface antigen (HBsAg), antibody to HBsAg (anti-HBs), hepatitis B e antigen (HBeAg), antibody to HBeAg (anti-HBe), and antibody to hepatitis B core antigen (anti-HBc). This panel of biomarkers will allow categorization of the women as HBV-immune, HBV-infected or HBV non-infected and non-immune whereby further clinical interventions, including vaccination for women who are non-infected and non-immune can be planned. The Group recommends that the minimum biomarkers should include HBsAg and HBeAg. If test results indicate that HBsAg is negative, women should be counselled for their result and receive the usual standard of care for pregnant women. If pregnant women are found to be HBV-infected (HBsAg positive), the woman should be jointly managed by the obstetrician for the wellbeing of the fetus and woman, and managed for her hepatitis disease status with the hepatologist/gastroenterologist or infectious disease specialist during pregnancy,

labour, delivery and postpartum. Continued follow-up after delivery will be required.

2. Initial assessment (baseline) and management decisions

The Group recommends staging of liver disease status for HBsAg positive pregnant women to determine the degree of liver injury. Baseline tests include maternal HBV viral load (HBV DNA), HBV serologic makers, liver function test, and upper abdominal ultrasound. Management decisions should consider the following:

2.1 In HBeAg positive pregnant women with HBV DNA > 20,000 IU/mL or HBeAg negative with HBV DNA > 2,000 IU/mL and significant hepatic activity, alanine aminotransferase (ALT) ≥ 5 × upper limit of normal (ULN) (excluding other potential etiologies), or liver cirrhosis, the consideration to start antiviral therapy is for the woman's health. The drug of choice is TDF which should be prescribed after clinical assessment, counselling, patient education and informed consent by specialists in hepatology or infectious diseases. The decision to start antiviral treatment and patient management plan should be communicated to the obstetric team.

2.2 In pregnant women with positive HBV DNA and ALT ≥ 2 to < 5 × ULN, antiviral therapy can be postponed. Close monitoring based on clinical symptoms and serology is required throughout the pregnancy. The Group recommends at least liver function monitoring every four weeks during pregnancy. If ALT increases to ≥ 5 × ULN, treatment decisions should follow point 2.1; if ALT reduces to < 2 × ULN, treatment management should follow point 2.3; if ALT remains within the range ≥ 2 to < 5 × ULN, consider starting antiviral treatment at gestational week 24 using TDF after patient education and informed consent.

2.3 In pregnant women found to have viraemia, ALT < 2 × ULN, and without cirrhosis, there is no indication to initiate antiviral therapy. Clinical and liver function monitoring is recommended. If, there are signs of progression of liver disease, that is, ALT ≥ 2 × ULN during the course of pregnancy, treatment considerations should follow point 2.1 or 2.2. accordingly. In addition, total bilirubin (TBIL) and prothrombin activity (PTA) should be tested to evaluate the severity of liver disease.

3. Management during pregnancy

In pregnant women with normal or slightly elevated ALT, HBV DNA should be quantified with sensitive real-time polymerase chain reaction (PCR) method in the second trimester. In order to prevent MTCT of HBV, antiviral treatment should be considered based on HBV DNA quantification.

3.1 If HBV DNA is above 2×10^5 IU/mL, antiviral treatment with either TDF or LdT should be administered after patient education and informed consent at gestational week 24-28.[3] In pregnant women with very high baseline viral load (≥ 1×10^8 IU/mL), earlier initiation of antiviral therapy may be necessary to achieve viral suppression below recommended threshold.[3] Before delivery, HBV DNA levels should be repeated to evaluate efficacy of antiviral therapy and risk of MTCT.

3.2 If HBV DNA is < 2×10^5 IU/mL, antiviral therapy is not recommended, but follow-up should be performed during gestation.[3]

4. Delivery management

4.1 Mode of delivery: The delivery mode should follow the usual obstetric indications. Routine cesarean section is not recommended for the prevention of HBV transmission.

For cases of HBV MTCT, the large proportion occurs around labour and delivery, particularly with risk factors including high maternal viraemia, transfusion of the mother's blood to the fetus during labour contractions, infection after rupture of membranes and direct contact of the fetus with infected secretions or blood from the maternal genital tract. At the delivery time, clinical assessment of HBV infected pregnant women should include the liver function status. Where possible and with consideration to the safety of the fetus, procedures which break the skin and mucosal barrier should be avoided as much as possible, and include fetal scalp electrodes, fetal scalp blood sampling and vigorous aspiration of oral suctioning of the baby at birth. Instrumental delivery such as vacuum extraction and forceps to expedite delivery during the second stage of labour, should follow obstetric indications. There is a small risk of traumatizing the fetal skin and risking transmission of HBV to the infant.

4.2 Care of the newborn: Standard precautions should continue when handling the newborn. Visible blood, mucus, and amniotic fluid, from the surface of the neonate and the cord should be gently wiped off. Standard procedures for cord cutting should be followed. Vaccines to be delivered at birth including the hepatitis B vaccine, immunoglobulin and vitamin injections should be provided. The skin at the injection rite should be cleaned with an alcohol swab before administering the injection.

5. Discontinuation of antivirals

For women who do not need antiviral therapy for her own health, drugs can be discontinued immediately after delivery.[3] Patient education to return if symptoms occur as well as regular monitoring of the ALT should be done post-partum, to detect

for post-partum hepatitis flares. For women who were started on antiviral therapy for her own health, treatment should be continued after delivery. The woman should continue care for her hepatitis status with hepatologists/gastroenterologists or infectious disease specialists. Discontinuation of the antiviral therapy can be considered following the recommendations in The Chinese Guideline of Prevention and Treatment of Chronic Hepatitis B (2015 Version).[3]

6. Neonatal immunoprophylaxis

6.1 Within 12 hours after delivery, HBIG 100 IU should be administrated as soon as possible intramuscularly in the anterolateral thigh or deltoid muscle.

6.2 At the same time, the first dose of the recombinant yeast hepatitis B vaccine (10 μg/ 0.5 mL) should be administrated on the opposite side in the anterolateral thigh or deltoid muscle. The other two doses of 10 μg/ 0.5 mL hepatitis B vaccines are scheduled at month 1 and 6 of age following the national Chinese vaccination schedule.

6.3 Delayed vaccination schedule: If the second dose of the hepatitis B vaccine was missed from the original schedule within 3 months of the initial planned visit, this dose should be injected as soon as possible, and the third dose of vaccine should be provided at month 6. If, however, the second dose is delayed more than 3 months of the intended planned visit, the second dose should be given as soon as possible and the third dose should be scheduled for 2 months later.

6.4 Preterm and low-birth-weight infants ($<$ 2000 g): HBIG 100 IU plus hepatitis B vaccine (10 μg/ 0.5 mL) should be administrated within 12 hours in this group, with

other 3 doses of subsequent vaccine (10 μg/ 0.5 mL) scheduled at 1, 2 and 7 months of age. Infants with unknown maternal HBsAg status should still be treated as infants born to mothers who are HBsAg-positive. Their mothers should be tested for HBsAg as soon as possible. If maternal HBsAg is negative, the three doses of vaccine could be started at 1 month of age or be postponed until just before hospital discharge. The vaccination scheme should still be administered according to 0-1-6 schedule.

7. Breastfeeding

7.1 Mothers who were not treated with antivirals during pregnancy are encouraged to breastfeed as long as their newborns have received combined immunoprophylaxis composed of HBIG and hepatitis B vaccine. If hepatitis flare occurs during breastfeeding postpartum, mothers could be managed referring to Chinese Guideline of Prevention and Treatment of Chronic Hepatitis B (2015 version).[3]

7.2 For mothers on antiviral treatment as prevention for MTCT, antiviral treatment can be discontinued after delivery, and then breastfeeding is allowed if newborns received combined immunoprophylaxis.

7.3 If mothers receive antivirals for treating CHB and continued treatment after delivery, in such case breastfeeding is not contraindication.

8. Follow-up of infants

Infants should be followed up following the usual post-partum standards for neonatal care.

9. Post-vaccination serologic testing (PVST) for the HBV-exposed infant

WHO recommends PVST for infants born to HBV-infected pregnant women. PVST

should be performed after completion of the hepatitis B vaccine series and at least 1 month after last hepatitis B vaccine dose. The PVST consists of two markers, HBsAg and anti-HBs (antibody levels) , which will allow determination if these infants are immune and protected, infected or non-infected and non-immune. Quantification of anti-HBs antibody level will help determine protective levels, defined to be at the threshold of ≥ 10 mIU/mL.

9.1 HBsAg-positive infants should be referred for appropriate clinical care and follow-up and their parents counselled.

9.2 For infants found to be HBsAg-negative and with anti-HBs ≥ 100 mIU/mL, they are hepatitis B protected and do not require further medical management.

9.3 For HBsAg-negative infants with anti-HBs < 10 mIU/mL, these infants should have HBV DNA tested to rule out the possibility of occult HBV infection. If HBV DNA is undetectable, these infants should be re-vaccinated with a further three doses of hepatitis B vaccine (10 μg/0.5 mL), and undergo PVST 1-2 months after the final vaccine.

9.4 Protective levels of anti-HBs: An antibody level of > 100 mIU/mL indicates a good immune response with protective immunity, while an antibody level of less than 10 mIU/mL indicates no significant response or immunity. An antibody level of 10-100 mIU/mL following vaccination indicates intermediate immunity.

10. Follow-up of mothers after delivery

10.1 Mothers on antiviral treatment after delivery should be followed up per protocol for general CHB patients. Liver functions and HBV DNA should be checked every 3

months. HBV serology, alpha-fetoprotein (AFP), abdominal ultrasound, and transient elastography should be performed at 6-monthly interval.

10.2 Liver functions and HBV DNA should be checked at 6-8 weeks after delivery in mothers who discontinue antivirals after delivery. If the liver functions are normal, monitoring should be continued at an interval of 3-6 months. If liver function is abnormal at 6-8 weeks after delivery, patients should be managed following the recommendations in Chinese Guideline of Prevention and Treatment of Chronic Hepatitis B (2015 updated version).[3]

10.3 Mothers not on antiviral treatment may experience ALT flare or exacerbation after delivery. Liver function should be monitored at postpartum weeks 3-4 and 9-12, especially in those with elevated ALT and detectable HBV DNA level at delivery.

References

1. Hou, J. et al. Management Algorithm for Interrupting Mother-to-Child Transmission of Hepatitis B Virus. *Clin Gastroenterol Hepatol.* **17**, 1929-1936 (2019).

2. National Health and Family Planning Commission of the People’s Republic of China. Action plan to prevent mother-to-child transmission of AIDS, syphilis, and hepatitis B (2015). http://www.nhfpc.gov.cn/ewebeditor/uploadfile/2015/06/20150615151817186.docx. Accessed June 2015.

3. Hou J, Wang G, Wang F, et al. Guideline of prevention and treatment for chronic hepatitis B (2015 update). *J Clin Transl Hepatol.* **5**,297-318 (2017).

Research protocol

A Real World, Multi-center Study on Mother-to-child Transmission of Hepatitis B Virus in China (SHIELD Program)

**Study Initiator:**	Chinese Foundation for Hepatitis Prevention and Control
	Nanfang Hospital, Southern Medical University
**Principal Investigator:**	Jinlin Hou
**Document type:**	Clinical research protocol
**Version number:**	2.2
**Version date:**	June 16, 2015

All information in this document belongs to Nanfang Hospital and Tiger Medical Co.Ltd., which is strictly confidential and prohibited to be disclosed to any third party without permission in written from Nanfang Hospital and Tiger Medical Co.Ltd. With authorization of Nanfang Hospital, the document is not allowed to be photocopied, stored in any retrievable system or transferred in any ways, electronically or other forms. It should be submitted to Nanfang Hospital and Tiger Medical Co.Ltd in a timely manner, as appropriate.

CONTENTS

1. INTRODUCTION 3
2. BACKGROUND AND RATIONALE 4
3. ENDPOINTS 7
3.1 PRIMARY ENDPOINTS 7
3.2 SECONDARY ENDPOINTS 7
4. DESIGN OF THE STUDY 7
5. TARGET POPULATION 7
5.1 INCLUSION CRITERIA 8
5.2 EXCLUSION CRITERIA 8
6. METHODOLOGY 8
6.1 STUDY TOOLS 8
6.2 DATA COLLECTION 8
6.3 LAB TESTS AND SPECIMEN COLLECTION 9
6.4 IDENTIFICATION OF SUBJECTS 10
6.5 INTERVIEWS 10
6.6 DEFINITIONS 11
7. STATISTICAL ANALYSIS 11
7.1 DATA ANALYSIS 11
7.2 SAMPLE SIZE 11
8. DATA ASSESSMENT AND DATABASE MANAGEMENT 12
8.1 RESEARCH CENTER SUPERVISION 12
8.2 DATABASE MANAGEMENT AND QUALITY CONTROL 13
9. RESPONSIBILITIES OF INVESTIGATORS 13
9.1 ETHICAL CONSIDERATIONS 13
9.2 BENEFITS OF SUBJECTS 14
9.3 PROCESS AND DOCUMENT OF INFORMED CONSENT 14
9.4 MODIFICATION OF PLAN 14
9.5 TERMINATION OF PROJECT 15
9.6 DATA SECURITY 15
9.7 MONITORING 15
9.8 REVIEW 16
9.9 DATA RETENTION 16
9.10 DISSEMINATION AND PUBLICATION 16
10. TIMELINES 16
11. INVESTIGATORS/LOCATIONS 16
12. REFERENCES 27

1. Introduction

**Project title**	A Real World, Multi-center Study on Mother-to-child Transmission of Hepatitis B Virus in China
**Principal Investigator**	Jinlin Hou; Nanfang Hospital, Southern Medical University
**Project location and subject population**	Location: China<br>Subject population: pregnant women with chronic hepatitis B (hereinafter referred to as "HBV pregnant women"; see inclusion criteria and exclusion criteria)
**Project period**	July 2015 – December 2025
**Endpoints**	**Primary endpoints:**<br>Rate of mother-to-child transmission of HBV<br>Rate of birth defect<br>**Secondary endpoint:**<br>Observe the change of HBV DNA level in mothers with antiviral therapy from baseline to withdrawal of antiviral drugs
**Project design**	Two multi-center, prospective cohorts: 1) Nationwide hospital-based cohort ; 2) Community-based cohort (Bao' an district, Shenzhen)
**Sample size**	Plan to recruit 50,000 HBV pregnant women
**Inclusion criteria**	Pregnant women with chronic HBV infection
**Exclusion criteria**	Positive serologic test for human immunodeficiency virus or hepatitis C virus;<br>Any co-morbidity that might reduce compliance;<br>Unable or unwilling to use the mobile health application-Shield APP.
**Follow up time**	HBV-infected mother-infant pairs would been followed to post vaccination serological test.
**Assessment method**	Collect data of pregnant women and their infants during and after pregnancy; calculate incidence rate of mother-to-child transmission and incidence rate of birth defects among infants.

**Main measurements**	**Pregnant women**<br>HBV related indicators<br>◆ HBV serological markers (HBsAg, HBsAb, HBeAg, HBeAb and HBcAb)<br>◆ HBV DNA<br>◆ Liver function<br>Other related indicators<br>◆ Demographic data<br>◆ Antiviral treatment history<br>◆ Pregnancy and labour history<br>◆ Co-morbidity<br>◆ Mode of delivery<br>◆ Breastfeeding<br>**Infants**<br>HBV related indicators<br>◆ HBV serological markers (HBsAg, HBsAb, HBeAg, HBeAb and HBcAb)<br>◆ HBV DNA<br>◆ Liver function<br>Developmental indicators<br>◆ Neonatal characteristics (height, weight, head circumference, Apgar score and any major birth defect)
**Statistical analysis**	Continuous variables are presented as the mean (± standard deviation), and categorical variables are presented as percentages. Categorical variables were analysed with Pearson's chi-square test or Fisher's exact test. Univariable logistic regression analysis was used to identify clinically relevant variables associated with MTCT in stage II. Collinearity diagnostics was conducted and variables showing a $p<0.10$ in univariable logistic regression analysis were entered into multivariable logistic regression model. All statistical tests were two-sided. A P value <0.05 was considered statistically significant.

2. Background and rationale

HBV infections tend to be prevalent globally. According to the WHO, 2 billion people are infected with HBV, and 350 million people are CHB patients. Each year, some 1 million people die from HBV-related liver failure, cirrhosis and hepatocellular carcinoma[1,2]. China is a highly epidemic hepatitis B area. Since 1992, when the hepatitis B vaccination programme was introduced, the HBV infection rate has declined dramatically. According to the 2006 National Epidemiological Survey of

Hepatitis B, the HBsAg carrier rate of the population aged 1-59 was 7.18%[3]. While preventive measures are quite effective, the HBsAg carrying rate is still 0.96% among children under age 5[4]. It is estimated that among the infants born to pregnant women with HBV viremia, approximately 8%-15% of vaccinations against HBV are ineffective, resulting in HBV mother-to-child transmission[5-7]. Given the large population of China, the 0.96% HBsAg carrier rate remains a serious public health problem for the future. After the introduction of an HBV vaccination programme and enhancement of blood and blood product safety management, mother-to-child HBV transmission has now become the main mode of transmission. Epidemiological evidence shows that HBV DNA load in the peripheral blood of pregnant women is an independent risk factor for HBV mother-to-child transmission[5,7,8]. When the HBV DNA load in the peripheral blood of pregnant women is over 10^6 copies/ml, the incidence of HBV mother-to-child transmission significantly increases. Domestic and international studies have shown[7-10] that taking anti-HBV nucleoside drugs during pregnancy can significantly reduce the HBV mother-to-child transmission rate. Han et al. studied the effectiveness of taking telbivudine during 20-32 gestational weeks in blocking HBV mother-to-child transmission. Among 135 HBV positive pregnant women who took telbivudine during late pregnancy, there was no HBV mother-to-child transmission, whereas in the control group who did not take telbivudine, the HBV mother-to-child transmission rate was 8%, indicating that taking telbivudine during late pregnancy can effectively block HBV mother-to-child transmission[7]. This was later confirmed by studies by Zhang Hua, Beijing You An Hospital and American researchers Pan CQ et al.[8,10]. In view of the efficacy and safety of telbivudine used during pregnancy, APASL[11] and EASL[12] now recommend nucleoside drugs (tenofovir, lamivudine and telbivudine) to be used during pregnancy to treat HBV infection and block HBV mother-to-child transmission.

Although some progress has been made recently in blocking HBV mother-to-child transmission, we have recognized the following issues in this area: 1) There are still significant gaps between clinical practice and clinical trials. Subjects in clinical trials

are special populations that are under standardized management; however, in China, there are not yet standard operable guidelines for the management of HBV pregnant women during pregnancy, and doctors' perceptions and understanding of this issue also vary. There are also great disparities in healthcare services among different regions in China. All of these pose challenges to efforts to block HBV mother-to-child transmission. 2) Baseline epidemiological data of pregnant HBV women are lacking. There are no systematic epidemiological studies of HBV pregnant women, leading to the lack of a good understanding of the virological and clinical features of HBV pregnant women during pregnancy. 3) The issue of when to stop the prophylactic use of antiviral drugs during pregnancy for HBV pregnant women has not been resolved. It is not clear what is the optimal timing for terminating the prophylactic use of antiviral drugs during pregnancy and whether viral hepatitis will bounce back. 4) There is a lack of long-term safety evidence on the impact of nucleoside drugs on fetuses and infants. Previous studies of HBV mother-to-child transmission involved prophylactic use of nucleoside antiviral drugs in late pregnancy; these studies had small sample sizes. There is still no safety data on the effects of taking these drugs in the early and intermediate terms of pregnancy among the Chinese population. An antiretroviral pregnancy register system was established in the 1990s in the US (antiretroviral pregnancy register, APR), which provides valuable evidence on ARV's safety during pregnancy; however, the data were mostly on HIV with little on HBV; the effects on HBV in such studies in China are still not known.

In this regard, we believe it is necessary to conduct a prospective epidemiological study of pregnant HBV women to understand the rate of mother-to-child transmission and the safety of using antiviral drugs during pregnancy. The findings can provide a basis for evidence-based effective management of pregnant HBV women to reduce the HBV mother-to-child transmission incidence rate, guide safe medication use among HBV women for the prevention and treatment of hepatitis B during pregnancy, and provide evidence for policy makers in developing relevant policies.

3. Endpoints

3.1 Primary endpoints

- Rate of mother-to-child transmission of HBV;
- Rate of birth defect.

3.2 Secondary endpoints

- Observe the change of HBV DNA level in mothers with antiviral therapy from baseline to withdrawal of antiviral drugs.

4. Design of the study

The study is a prospective cohort study with HBV pregnant women as the target population.

HBsAg positive pregnant women are selected as the subjects; eligible HBV pregnant women are recruited for prospective observation. The study includes documentation of liver functions, viral load changes and medication during pregnancy and 6-8 weeks after childbirth. Prospective observation of infants to document the status of HBV vaccination, liver functions and HBV indicators of the infants from birth to 7-12 months of age is continued. In the study, no research driven treatment will be provided to the subjects; the decision regarding whether to treat and what treatment options to choose will only be made by the attending doctor based on the medical conditions and the willingness of the subject.

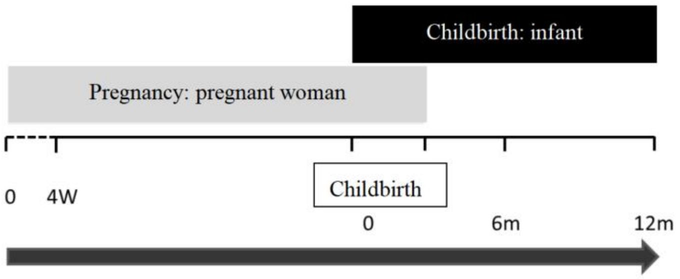

Figure 1: Study design

5. Target population

The target population is pregnant women with CHB (hereafter referred to as "HBV pregnant women"). The planned recruitment is 50,000 HBV pregnant women, including HBeAg positive and HBsAg negative women.

5.1 Inclusion criteria

All of the following inclusion criteria must be met:

– Pregnant women with chronic HBV infection.

5.2 Exclusion criteria

Pregnant women with any of the following are excluded:

– Positive serologic test for human immunodeficiency virus or hepatitis C virus;
– Any co-morbidity that might reduce compliance;
– Unable or unwilling to use the mobile health application-Shield APP.

6. Methodology

6.1 Study tools

Mobile health application – "SHIELD" App: The "SHIELD" App is designed and developed for pregnant HBV women and is used in the project for follow-ups and management of pregnant HBV women during and after pregnancy, including for data collection, transfer, doctor-patient communication and patient education, improving patients' awareness of diseases, and patient compliance.

6.2 Data collection

6.2.1 Registration and baseline data: the following should be ensured during the recruitment of HBV pregnant women:

– All inclusion criteria and exclusion criteria are met
– Informed consent letter is signed
– Clinical diagnosis is consistent with CHB (HBsAg positive more than 6 months)
– Demographic data
– Antiviral treatment history
– Pregnancy and labour history
– HBV serological markers (HBsAg, HBsAb, HBeAg, HBeAb and HBcAb)
– HBV DNA
– Liver function

6.2.2 Assessment at the 28th week of gestation

Tests include the following:

- HBV DNA
- Liver function
- Antiviral treatment

6.2.3 Assessment at delivery

- Outcome of pregnancy (live birth, still birth)
- Delivery mode (normal, C-section)
- Neonatal characteristics (height, weight, head circumference, Apgar score and any major birth defect)
- HBV DNA
- Liver function
- Antiviral treatment
- Infant vaccinations

6.2.4 Post-partum follow-up at 6-8 weeks after delivery

- Infant vaccinations
- Breastfeeding
- HBV DNA
- Liver function
- Antiviral treatment

6.2.5 Post-partum follow-up 2 (infants between 7-12 months of age)

- Infant vaccinations
- HBV DNA
- Liver function
- Antiviral treatment

6.3 Lab tests and specimen collection

◆ Key items:

HBV indicators (HBV indicators and quantitative HBV DNA)

HBV indicators (HBsAg, HBsAb, HBeAg, HBeAb, HBcAb): electrochemiluminescence immunoassay

quantitative HBV-DNA: fluorescence quantitative PCR

◆ Specimen collection:

Pregnant women: 5 ml plasma and 5 ml whole blood at 26-28 weeks of gestation (for genomic tests in the future); 5 ml plasma at childbirth

Infants: 5 ml serum at 7-12 months

Collect placental blood (placental HBV infection) and umbilical blood (placental and umbilical blood are collected in some hospitals) at childbirth.

6.4 Identification of subjects

Each recruited subject has a unique code (subject code) that is referred to throughout the project. The subject code consists of 6 digits: XBK-XX-XXXX:

- XBK indicates project name;
- Digits 1-2 indicate the research centre;
- Digits 3-6 indicate the serial number of the subject, e.g., 0001 is the first subject recruited and 0002 is the second subject recruited.

6.5 Interviews

A meeting can be convened before enrolling the subjects, as appropriate, to ensure that the investigators can correctly inform the subjects and/or legal guardians of the objectives, significance, contents, process, benefits and risks of the project. All materials or documents used for the meeting should be submitted to the Ethical Review Committee for approval before the start of the project.

Investigators and/or field investigators should:

- Inform the subject or parents or legal guardian of the relevant information of the project (goals and objectives, design, steps, etc.) and answer any questions they raise to ensure the subject or legal guardian understands all information in the informed consent letter;
- Acquire signed (written) informed consent from the subject or legal guardian;
- Assign a code to the recruited subject;
- Verify whether the subject meets all inclusion and exclusion criteria and record it on the CRF form;
- Record the subject's information on the CRF form and in the App according to the

requirements in the research protocol. All data should be kept confidential and correctly stored;

- Blood specimens should be collected from all subjects at 26-28 weeks of gestation; the blood will be used to understand HBV quantitative load. This test is free of charge.

6.6 Definitions

6.6.1 HBV mother-to-child transmission: The rate of HBV mother-to-child transmission is defined as: HBsAg positive is found in the peripheral blood of infants at 7-12 months of age who were born to HBV pregnant women and have completed HBV vaccination.

6.6.2 Congenital defects: refers to the morphological or structural abnormality an infant carries at birth.

6.6.3 Apgar score: assessment and score for the infant based on skin colour, heart rate and pulse, respiratory rate, muscle tone and movement, and reflexes. Newborns who score 10 points are normal; those with scores below 7 points have mild asphyxia; and those with scores below 4 points have severe asphyxia.

7. Statistical analysis

7.1 Data analysis

Continuous variables are presented as the mean (± standard deviation), and categorical variables are presented as percentages. Categorical variables were analysed with Pearson's chi-square test or Fisher's exact test. Univariable logistic regression analysis was used to identify clinically relevant variables associated with MTCT in stage II. Collinearity diagnostics was conducted and variables showing a $p<0.10$ in univariable logistic regression analysis were entered into multivariable logistic regression model. All statistical tests were two-sided. A P value <0.05 was considered statistically significant.

7.2 Sample size

This study is an observational study without estimation of sample size. The plan is to enroll 50,000 HBV positive pregnant women, including both HBeAg positive and

HBsAg negative subjects.

8. Data assessment and database management

8.1 Research centre supervision

Informed consent must be obtained from each subject or his/her legal guardian before the start of the project and enrolment of the subject. Each investigator should keep the informed consent letter with the original signature. A photocopy of the signed informed consent letter will be given to the subject. Researchers will only start the activities required in the research protocol after valid informed consent has been obtained. The date that informed consent was obtained must be recorded in the e-CRF and original documents. If the research protocol is revised, the informed consent letter should also be revised to reflect the changes. After approval by the Ethical Review Committee, the revised informed consent letter must be signed by the participating subjects and the potential subjects before they participate in the project.

During the project process, the local supervisors will visit the research centres regularly to check the completeness of the records of subjects, the accuracy of the e-CRFs, the research protocol, and the progress of research registration. The key investigators should provide assistance to the supervisors during the process.

Researchers must keep the original documents within the research centre, which include case records and visit records (hospital or clinical records); visit records include demographic data, medical information, research department data and any other test or assessment findings. All information in the subjects' e-CRF must be traceable to the original documents. It should be clearly defined before the start of the project which data do not need hard copy records and can exist only in the e-CRF. Researchers must keep the original informed consent signed by the subject (a photocopy is kept by the subject).

Researchers should make all original documents available to supervisors for review to check for consistency with the information in the e-CRF. The following are required to be checked: keeping the informed consent letters; compliance with the inclusion/exclusion criteria; and recording data on key variables and safety variables.

According to the established supervision plan, supervisors will also conduct supplementary auditing to compare the original data with the data in the e-CRF. Identification information of the subject in the original documents should not be disclosed publicly.

8.2 Database management and quality control

Data in case reports will be entered into the database by contract research organization (CRO) staff according to their internal standard operating procedures.

The data management group was responsible for converting the data uploaded to the SHIELD APP to digital information. Data managers will conduct systematic data reviews based on automatic verification procedures and error information produced by the database sheets. Ten percent of laboratory test reports were routinely randomly monitored every week. Significant errors are corrected by data managers; other errors or omissions should be entered into the data correction form and the form should be returned to the research centre for correction. Signed original forms and data correction forms should be kept within the research centre so that the corrected data can be entered into the database. All key data on safety and effectiveness should be reviewed for quality control before the database is locked. Medical history/current history and adverse events should be coded according to clinical research data coding conventions. The specimens for HBV testing and quantitative HBV DNA testing should be centralized, treated and disposed of; the results should be transferred electronically to the data processing centre. Missing data were retrieved via the SHIELD APP and telephone follow-up.

After the above work is performed and the database is declared to be complete and accurate, the database will be locked. Any further modifications to the database must be approved in written form by both the CRO and the project chief.

9. Responsibilities of investigators

9.1 Ethical considerations

The research project is conducted strictly according to the revised *Edinburgh Declaration of Helsinki*, which was formulated by the International

Pharmacological-Epidemiological Association in Nov 2007 as the guidance for epidemiologic studies (US Epidemiological Guidelines for Pharmacokinetics and Vaccine Research) (http://www.dundee.ac.uk/iea/GEP07.htm).

Before the first subject is recruited, the research protocol must be approved by the Ethical Review Committee. The researcher/research institute and the representatives of participating units sign the agreement. Consensus regarding financial aspects and division of labor among the parties should be reached before the official start of the project.

9.2 Benefits of subjects

In this study, the "SHIELD" app will facilitate communication and interaction between pregnant women and doctors and provide pregnant women with medical information through regular updates of the knowledge base to improve pregnant women's awareness of the disease and improve compliance to promote maternal and child health.

9.3 Process and documentation of informed consent

Investigators are obligated to inform the subject or his/her legal guardian in detail regarding the information related to the project and to obtain informed consent. If the subject or his/her parent/legal guardian agrees to participate after understanding the nature of the project, an informed consent letter (in duplicate with one copy each kept by the parent and the investigator) should be signed before the participation of the subject. The investigator should have a full understanding of the updated information in the informed consent letter. The information in the informed consent letter should be presented in the local language.

9.4 Modification of the plan

No revision of the protocol is allowed before approval by Nanfang Hospital and Tiger Company. If modification of the agreement is necessary during the project, discussion will be conducted between Nanfang Hospital and Tiger Company. If both agree to the modification after discussion, Tiger Company will make the modifications in written form that will become part of the official agreement.

Any modification to the project plan contents should be approved by the Ethical Review Committee.

Modification of project management refers to adjustments to the management and logistics of the project, which should not harm subjects' safety; it also refers to modification of the goals and objectives and progress of the project. Such modifications do not require approval from the Ethical Review Committee.

All modifications should be implemented as planned without approval from the Nanfang Hospital Department of Infection Control and the Ethical Review Committee, unless the health of subjects will be harmed.

Any modifications to the research protocol should be documented in the research agreement of the two parties.

9.5 Termination of the project

The project may be terminated due to special reasons and/or the decisions of Nanfang Hospital and/or the Ethical Review Committee. If the project is terminated or suspended in the early stages, Tiger Company and the Ethical Review Committee should be formally notified of the reasons for the termination or suspension.

9.6 Data security

After the start of the project, the lead unit and the participating units and Tiger Medical Co., Ltd. should keep all data confidential and should not disclose the contents, data and findings of the project to non-relevant third party and commercial competitors.

9.7 Monitoring

The project coordinator of Tiger Company and the epidemiologists should ensure the availability of research materials; investigators should understand the requirements and SOPs of the project.

The project coordinator of Tiger Company or his/her representative should routinely follow up on the project and should have access to the CRFs for review.

During supervision and follow-ups, the project coordinator of Tiger Company should have quality control over the project and should discuss specific operations with the

PI.

9.8 Review

Tiger Medical Co., Ltd. is in charge of quality control to ensure that the project is conducted in accordance with the research plan and protocol, and to ensure that research data can be referred to and reviewed as appropriate.

9.9 Data retention

The Infection Control Department of Nanfang Hospital is to keep relevant research materials and documents during the project period until the conclusion of the project (official submission of the final report).

9.10 Dissemination and publication

The data from this project are jointly owned by the investigators and Tiger Medical Co., Ltd. All intended publications or reports related to the project should be approved by the investigators and Tiger Company before submission. The investigators will summarize research findings and publish scientific articles in journals, both national and international, as well as present at workshops.

10. Timelines

Sep 2014 Draft project proposal

Oct 2014 APP design and development

Jan 2015 APP testing and online

Mar 2015 Seminar to discuss project plan

July 2015 Ethical review;Project launch;HBV positive pregnant women enrolled and followed up

June 2025 Enrolment and follow-up completed

December 2025 Data analysis; Publication

11. Investigators/Locations

**Guangdong Province**			
Peifen Guo	Hongjun Li	Xiafei Fu	Guowei He
Xiaozhu Zhong	Chunxiao Wu	Yuanping Zhou	Ying Deng
Yingju Li	Yunfei Gao	Muhua Li	Yonghua Huang
Jinlin Hou	Peng Wang	Youfu Zhu	Qun Zhang

Zhefan Dai	Dongmei Hu	Yaping Wang	Suihua Feng
Yanying Huang	Mei Zhong	Yingying Huang	Chuangneng Lao
Manhua Zhong	Wenjun Zhang	Jie Peng	Yuanqiang Yang
Yaoyong Zhou	Liujuan Li	Ronglong Jiang	Linlin Lu
Zhanzhou Lin	Suiwen Wen	Xueru Yin	Shiyao Xian
Wenyu Mo	Hongying Zhu	Jian Sun	Xiaolu Liu
Canhui Xiao	Suran Huang	Huaiyu Chen	Weiping Chen
Bo Wan	Hongling Chen	Xiaorong Feng	Jia Wang
Jing Li	Jianjun He	Fuyuan Zhou	Xiaohua Li
Yanyan Yin	Lei Xiao	Weiqun Wen	Yan Tan
Qing Shan	Min Xu	Yongpeng Chen	MingcongZhao
Yurong Chen	Liling Li	Jinjun Chen	Changzheng Hu
Xiaoyan Li	Feifei Huang	Li Liu	GangHe
Huihua Liao	Yuyan Bai	Jinzhang Chen	Yueying Zhen
Weidong Luo	Yinong Ye	Xiaoyun Hu	Shaoqun Liang
Huiyuan Liu	Yuanling Xiao	Dingli Liu	Ming Luo
Biyan Liang	Xingliu Wu	Qiyuan Tang	Heming Wu
Hongshun Fan	Ming Chen	Dongying Xie	Xiaoqing Wang
Shu Yang	Li Liang	Zhenghua Ma	Zhijun Qu
Shilei Pan	Yu Quan	Xuemei Liang	Guimei Huang
Yingxia Liu	Yaping Lu	Honglian Bai	Daoyan Zhao
Suiqun Guo	Sujun Zhu	Caiqiue Cai	Hang Zhang
Youming Chen	Zhihong Liu	Jin Li	Jinyu Xia
Yingchun Li	Yuehua Chen	Jing Yuan	Xiaomou Peng
Yaotian Li	Pei Zhou	Xunhua Zhong	Fengyun You
Zheng Li	Keng Chen	Feijian Ao	Li Ding
Qixia Li	Qian Zhao	Simin Yao	Zhongsi Hong
Chenhong Wang	Yujie Li	Hong Yu	Chunna Li
Hong Yang	Yangyang Hu	Yanfeng Wang	Huili Chen
Zhihua Liu	CaixiaWang	Xueying Ruan	Mingxing Huang
Huixiu Zheng	Taojin Zeng	Lijuan Xiao	Jian Liu
Chuangguo Yang	Guiyu Gong	Haipeng Zhu	Xi Liu
Ling Song	Qingyu Li	Guixuan Chen	Zhaojuan Su
Qian Jiao	Jinfeng Ling	Jieqing Zhai	Jing Liu
Laiqin Peng	Mei Jiang	ZhongjunLi	Hongjun Sun
Yulan He	Yiping Luo	Wen Huang	Qiwen Yuan
Wenjian Li	Qi Zhang	Wanhua Wu	Niannian Chen
Songmei He	Yuanyuan Wang	Yuqing Li	Le Chen
Kewei Zhu	Yabing Guo	Lan Tang	Qi Li
Bei Zhong	Qinjun He	Chenhua Zhang	Ting Hong
Peng Zhang	Jinfang Zhou	Youming Chen	Miao Wang
Huafang Wang	Tianhuang Liu	Xiaohong Zhang	Yuanqiao Cheng
Ling You	Lihong Deng	Yongyu Mei	Wanjing Huang

Fan Yang	Zongyun He	Jing Lai	Yiping Zeng
Jun Wu	Zhancheng Yao	Jianguo Li	Qing Xu
Fangfang Zheng	Peishan Chen	Hongying Hou	Linli Sun
Jie Song	Xuan Zhou	Shuisheng Zhou	Yanru Lan
Zhizhong Deng	Xuan Li	Jianhui Fan	Youyuan Zhu
Youyou Wang	Zhifeng Chen	YuzhuYin	Shiwu Ma
Mo Chen	Xiaoxia Zheng	Zhenyan Han	Chong Zheng
Yangbin Guo	Ke Luo	Shibin Xie	Yuewen Guo
Sichun Yin	Congcong Li	Zhixin Zhao	Jianmin Luo
Yanzhong Peng	Zhenchang Wang	Hong Deng	Jiemei Liang
Guoxin Hu	Yue Su	Jianyun Zhu	Hongyu Zhang
Jing Wu	Lifa Zhang	Xuejun Li	Mingjing Lu
Lijia Chen	Rui Zhang	Lin Yang	Zhen Hu
Minghua Qi	Jun Xu	Yufeng Zhang	Lang Ming
Xindeng Tong	Xiaohua Chen	Yuehua Huang	Yong Huang
Ting Liu	Minfeng Liang	Chenghui Huang	Xiaoqiao Chen
Xiaohui Min	Hui Long	Aiqiang Zhou	Zhiqiao Zhang
Ruilie Chen	Ruixing Zhong	Chengyu Jiang	RongguoWang
Shaorui Lin	Qingyang Zhong	Cuiyu Feng	Yeqiong Zhang
Dinggang Zheng	Zongliang Li	Xinhua Li	Liang Peng
Xiangming Xiao	Peiqing Huang	Lifan Zhuang	Genglin Zhang
Lingjie Wu	Rufang Chen	Suqin Gan	Yanhui Ning
Haisheng Zhang	Haiming Yan	Xiuzhong Zeng	Zide Zhao
Genglong Guo	Wenjuan Tan	Shiliang Zhong	Jing Ma
Daqiao Zhou	Zhijian Yu	Zonghua Rao	Shaojun Zhu
Hui Gao	Duoyun Li	Xinzhi Zhang	Chunyan Lai
Yingjun Zheng	Jiong Yang	Jianhua Tian	Songmei He
Mei Qiu	Qin Yan	Zhemei Huang	Zhongbin Lin
GuilongZhuang	Yurong Wang	Hong Wang	Jinhong Yu
Ping Liu	Bing Bai	Lin Xiao	Yihong Chen
Yiqi Liang	Zengyou Liu	Xuegang Wang	Haidan Yang
XuliFu	Lin Zong	Qingmei Fu	Huizhong Kang
Guohang Li	Zhan Yang	Yao Liu	Xiaodan Zheng
Xi Yu	Peiyan Xu	Yingzi Long	Xiaoyi Fu
Jinying Mo	Boping Zhou	Yuejun Pan	Qingxian Cai
Jianyu Kuang	Guangdong Tong	Yuwei Tong	Xuanqiu He
Zhangjie Zhang	Fenghao Zeng	Xiaohong Ouyang	Hongbo Qin
Guoyu Tan	Lubiao Chen	Qiuli Xie	Li Zhuo
Yulin Zhan	Bingliang Lin	Guotao Lyu	Xiuhan Yang
Wei Zhao	Chaoshuang Lin	Lijiang Jiang	Jianxin Tan
Guoqiu Lin	Jing Liu	Chunrong Huang	Yongyin Li
Yiping Luo	JieZheng	Junchao Qiu	Xin Wei
Meijie Shi	Ping He	Chengwei Zhou	Jie Dong

Jiayi Xie	Fang Guo	Keli Yang	Qing Yang
Pu Wang	Huiqian Zeng	Damei Zhou	Yanhong Chen
Danchun Cai	Wanqing Ji	Wenting Zeng	Qian Li
Jia Shi	Yuewei Jiang	Zhihui He	Dan Chai
Huaichang Liu	Shufang Yao	Ling Yang	Xiaohui Liao
Miaogen Li	Hongbo Gao	Jing Shi	Yu Li
Xinghai Zhao	Miaoxin Lu	Hao Zhang	Hong Sun
Haibo Lou	Yanqiong Liu	Maomao He	HaiqunXiao
Yumei Chen	Zhaodi Huang	Ruqing Zhao	Zhiyi He
Huijie Guo	Wei Wang	Qiwei Luo	Yaping Gan
Xihua Fu	Meiting Huang	Yanwen Xu	Jianxin Liang
Guobin Zhao	Jinna Li	Yanqiu Li	Huanlian Wen
Guocheng Liu	Wanzhen Chen	Qiling Huang	Xushuo Xie
Yiping Luo	Xiaoxia Huang	Jing Wu	He Zhang
Weijian Li	Wanmei Hong	Aiqin Yuan	Yingyan Qu
Zhen Guo	Yujie Ren	Cuixia Zhong	Xiaohan Huang
Yan Peng	Meilan Guo	Jinwei Guo	Zengwei Liang
Chunling Hu	Meiling Yin	Jing Zhou	Weiyuan Liu
Limei Fan	Yuhua Zhang	Yuqian Ren	Weiyuan Liu
LikunXu	Qianwen Yang	Peng Hu	Lihua Wu
Liqun Li	Lingfang Long	Zhixiang Zhang	Jianhong Xia
Changqing Lin	Jundong Li	Jianen Yang	Liqi Su
Liuyu He	Huiqing Gao	Baomin Yin	Shi Ouyang
Yan Zhang	Qing Zeng	Jihong Li	Fangming Liu
Xiaofei Lyu	Qianhua Zhang	Fengmei Jin	Shuangming Cai
Huihua Liu	Dandan Hua	Xiaohong Yang	Tingting Peng
Yiting Liu	Xia Cai	Changli Duan	Haijian Fan
Wenhui Ye	Meiling Zeng	Li Liu	Guanglin Li
Yunzhong Guo	Pin He	Yingting Chen	Lili Yang
Si Chen	Hongxia Guo	Chunyan Chen	Xuan Zhong
Guang Shao	Wenting Mo	Xiaoxia Li	Yu Liu
Jinbin Lai	Xiujuan Zhang	LinfengHong	Lina Wang
Xicai Wu	Fang Wang	Xiaoling Liu	Jinfeng Liu
Louyu Chen	Linjun Zhou	Hongxia Zhang	Qiong Liang
Huachao Mai	DanWu	Lili An	Shan Huang
Wenni Zhang	Haifei Luo	Weiying Dong	Danfeng Yu
Huanshun Xiao	Guoxin Liufu	Lihong Lin	
**Anhui Province**			
Qiuping Dong	Jiabin Li	Yunlan Chen	Duanduan Zhou
Qian Duanmu	Fei Su	Yun Wang	Hui Zhang
Hui Wang	Qian Su	Yeying Ding	Nana Ji
Linghua Tang	Heng Sun	Li Yu	Xiaosu Zhang
Fenghua Wang	Qiulin Sun	Chunling Mao	Yuchen Pan

Xiujuan Li	Lingling Xia	Wei Zhang	Xingxing Jiang
Feng He	Qinxiu Xie	Panpan Zhang	Huizhi Huang
Weishun Hou	Xihai Xu	Hui Xue	Xuelian Zhang
Huafa Yin	Ying Ye	Jinhua Zhang	Lebin Wang
Zhongjing Xia	Zhenhua Zhang	Yiqun Huang	Chuanfu Wang
Li Ma	Qingling Zhang	Xiuyan Chen	Ling Wang
Zhongsong Zhou	Ce Chen	Caimei Gu	Fengsheng Jia
Jun Cheng	Xuefeng Dai	Zhi Li	Chunlin Jiang
Xiaoping Jiang	Jiaqi Liu	Shuxia Cao	Jing Wang
Hongbin Li	Zhaogang Cheng		
**Beijing**			
Qian Bian	Hua Zhang	Lili Liu	Qinqin Wang
Ming Wang	Yunxia Zhu	Jinfeng Chu	Wenjuan Pu
Bo Li	Huili Liang	Xiufang Yu	Mei Shi
Xin Liu	Lai Wei	Mingliang Dong	Shuai Wang
Xiang Gao	Bing Zhu	Dandan Li	Juqiang Han
Ruihua Tian	Yinghui Yin	Wei Zhao	Jinhua Xiong
Mei Wang	Xia Liu	Haiwei Sun	Yongqiang Ren
Lingzhi Chang	Xiaohui Liu	Jingfang Ren	Wei Wen
Chong Zhang	Jinhua Wang	Li Li	Yiming Zhou
**Fujian Province**			
Qianguo Mao	Chuncheng Wu	Jing Dong	Xiaowen Chen
Jinmo Tang	Yue Chen	Jing Chen	Yixian Shi
Jiaen Yang	Manying Zhang	Youbing Li	Naling Kang
Chong Gu	Jiaji Jiang	Dawu Zeng	Shengtong Weng
Lijian Huang	Yueyong Zhu	Su Lin	Lei Zheng
Ying Zheng	Qi Zheng	Yihong Chai	Liqing Zheng
Huiqing Liang	Jia You	Jiumei Zhang	Lyufeng Yao
Xianqiong Gong	Min Wang		
**Gansu Province**			
Fang Wang	Xinren Zhou	Jia Wei	Yan Wang
Shuxian Feng	Shenghao Yun	Nina Wang	Lin Pan
Junfeng Kou	Ruili Chen	Qian Chen	Wenfan Li
**The Guangxi Zhuang Autonomous Region**			
Qian Guo	Shengping Yin	Chunlian Meng	Yuting Bao
Jianghong Chen	Minghao Qiu	Renguo Lei	Ren Chen
Cunli Nong	Ping Tan	Fen Li	Dan Xu
Xiling Mo	Huihui Wu	Shuzhen Wei	Jingjiao Lei
Yufang Luo	Qiufang Wei	Dongyun He	Jinqiu Huang
Tingting Li	Xuehan Peng	Hua Meng	Yelin Wei
Jing Guan	Yingwei Li	Qin Hu	Cuimin Wang
Jun Meng	Lianhua Pan	Qinghua Lu	
**Guizhou Province**			

Qianjun Ren	Xiaoqiong Gou	Quan Zhang	Xiaocui Yang
Lingling Ma	Yang Li	Tianyong Luo	Jiuqian Li
Yanbing Xiao	Liqian Yang	Yayun Wu	Xiulan Zhang
Li Zhang	Yun Long	Shuang Lu	Qixiang Li
Ya Han	Yuanhong Liang	Mao Mu	Li Zhang
Lixia Wu	Mingliang Cheng	Baofang Zhang	Xinhua Luo
Dingying Yang	JunWu	Qin Liu	Tianzhao Liu
Jisha Du	Jing Yang	Kaisheng Deng	Mingjuan Zhu
Benshan Peng			
**Hainan Province**			
Biao Wu	Li Shi	Ping Qiu	Yanteng Zhou
Furong Xiao	Hui Gao	Xiaozhen Xu	Jian Fu
Feng Lin	Guanghua Pan	Shiming Zhou	Xiuchun Zhang
Suoxian Chen	Wei Shen	Duyun Cai	Baiyu Pan
Tao Wu	Ying Wang	Yuanxue He	Jiao Wang
Xiaoli Fu			
**Hebei Province**			
Yang Yang	Jianxia Li	Bo Li	Ying Qin
Huanwei Zheng	Caiyan Zhao	Jian Wang	Changfen Wu
Xiuli Chen	Fang Liu	Xiaojun Liu	Wei Wang
Baoshen Zhu	Hongzhu Yin	Jingru Ma	Dongxiang Han
Suwen Li	Yadong Wang	Zhongfu Mo	Jinyu Gu
Jianping Xu	Luyuan Ma	Xiaolei Cao	Siyu Li
Hongxia Tian	Qian Zhao	Chunyan Yu	Cuili Yang
Lijuan Sun	Wei Wang	Jing Liu	Yuchan Zhao
**Henan Province**			
Heping Zhao	Hui Yin	Liujiang Bai	Peng Wang
Jia Shang	Huibin Ning	Lingju Wang	Shuai Guo
Yu Wang	Ling Jiang	Zhijie Mu	Xiandong Liang
Yi Kang	Guoqiang Zhang	Pan Zhang	Wenliang Zhou
Hewen Wu	Jujun Hai	Yuanliang Huang	Ying Feng
Chongshan Mao	Fang Wang	Zhenhua Wang	Guotao Li
Junfeng Wei	Fengqi Han	Shixi Zhang	Chunyan Zhou
Junping Liu	Yujie Tan	Peipei Wang	Lihua Zhang
Li Wang	Yalan Sun	Juan Li	Liantao Zhang
Wei Li	Yumeng Zhu	Zan Shi	HuanrongHou
Zhen Peng	Danyan Zhu	Kai Li	Fengxian Yu
Jinhuan Qin			
**Heilongjiang Province**			
Yujie Zhao	Jingnan Shao	Ming Mao	Long Zhao
Liying Zhu	Hao Zhang	Xuguang Zhu	Xiwei Gong
Lihua Zhong	Jie Chi	Yanhua Xiao	Hongyan Zhang
Yun Wang	Xuwei Qin	Haiyan Wang	Yanlei Zhang

Lei Yu	Ying Shen	Zhichao Shao	Jingting Bi
Xiaohua Yang	Changqing Li	Juan Du	Wenyan Gong
Baoling Lu	Yanqiao Shi	Li Yin	Hongxiu Zhao
Yu Cheng	Yuan Tian	Yanli Dong	Dan Ma
Hong Yao	Yanbo Wang	Lei Pang	Nannan Zhao
Jian Fan	Guojun Zhang	Wanming Zhu	Dandan Dong
Yuanyuan Wang	Zhiying Fan	Ying Liu	Jing Wang
Guimei Liu	Ying Dai	Mingjing Wang	Yuxiu Song
Shu Guo	Li Cao	Qiu Wang	Li Gao
Wei Zhang	Tianwei Liu	Xiuli Jia	Yanwei Xu
Jinping Zhang	Yue Shi	Lijing Wang	Zenghui Li
Tao Huang	Jing Han	Hongmei Cao	Wei Yu
Binghua Yang	Jing Liu	Wei Zhang	Jieqiong Hou
Ran Liu	XinZhang	Tiansheng Cao	Xiqiu Zeng
Sen Liu	Shanshan Yang	Yanxia Zhang	Yumei Zhang
Huan Qi	Shimin Wang	Ran Liu	Huaixiu Wu
Dan Zhang	Lihui Feng	Wei Hu	Bolin E
Jingwen Zhang	Wenhan Yang	Yanjuan Cheng	Qiu Fu
Jinmei Feng	Weiling Zhang	Yuhua Cui	Mingzhu Wang
Yan Yue	Liwei Ji	Guoli Yan	Wei Wu
Weiwei Li	Yunsong Zhang	Li Zhou	Wanming Zhu
Jinwei Li	Liping Yu	Lili Wang	Yu Zhang
Jiqing Li	Dongying Fu	Haiyan Liu	Chao Wei
Wei Guan	Mingjie Sun	Shanshan Chen	Litao Liu
Hongyan Gai	Qiulin Wang		
**Hubei Province**			
Shaonan Yan	Zhiyong Zhang	Wanjiang Zeng	Min Chen
Bin Deng	Xiaobei Chen	Zuobing Wang	Jing Zhang
Xiaojing Jiang	Fan Yang	Cuifang Zhang	Wenmin Fang
Futao Zhao	Shiyun Guan	Jianfeng Yuan	Liming Li
Ying Liu	Shundong Huang	Fan Zhou	Qian Yang
Junli He	Ling Feng	Ping Zhou	Deng Pan
Le Song	Dean Tian	Jiangang Wang	Liqiong Huang
Guiyue Shen	Deying Tian	Zefu Nie	Yuye Shen
Xin Wang	Zhengang Zhang	Jun Yu	Jun Zhu
Hanyun Yang	Wangxian Tang		
**Hunan Province**			
Jun Quan	Lihua Hu	Feng Yi	Ling Wang
Yan Huang	Xiangzhen Long	Chihua Liu	Yukun Huang
Jianping Xie	Xiaomin Wang	Yunhua Zhu	Congzhi Li
Yuanyuan Wang	Yulan Jiang	Xiaofen Li	Fei Liu
**Jilin Province**			
Nan Zhao	Shuqin Zhang	Yulin Chu	Ying Song

Weibing Tong	Jing Jiang	Wei Liu	Wei Li
Yuqing Yan	Chong Wang	Xi Chen	HuiChen
Ru Guo	Lishu Zhang	Yuhuan Wu	Yuting Wang
Guiqing Chen	Haiying Sun	Qiulian Li	Bo Sun
Chang Shu	Ming Wang	Quan Sun	Bo Wang
Hongmei Xu	Yanling Li	Min He	Yuxi Ma
**Jiangsu Province**			
Guorong Han	Li Xiao	Lihua Ye	Xiaoyun Zhang
Hongxiu Jiang	Xiuzhen Yang	Juanjuan Fu	Liping Wang
Sugui Cheng	Yang Li	Dongmei Wang	Qin Ding
Zhengru Zhang	Bian Wang	Yongyan Tang	Guangde Yang
Xiuhua Sun	Wei Wang	Chancong Gong	Li Li
Mei Luo	Libing Han	Genju Wang	Yan Liu
Mengmeng Du	Aiwen Geng	Xin Yue	Chunyang Li
Lili Yang	Meilong Shen	Xiaoxia Tang	Wei Yao
Yinling Zhao	Xinhua Bu	Yu Zhang	Xiaohong Guo
Junhua Wu	HezhuWang	Xiaofang Jiang	Li Gong
Li Jiang	Aihua Huang	Xueping Li	Yi Ding
Shaojun Wu	Haiyan Jiang	Huibing Sun	Shufen Bai
Xiaoxiang Wang	Yiqun Wu	Shasha Luo	Guifang Gu
Cen Xu	Shanshan Shao	Aihua Kong	Min Su
Jing Wei	Lihua Huang	Xiaomei Ding	Ji Li
Hongzhan Sun	Bo Zhang	Chuanwu Zhu	Lin Ye
Lin Wang	Shangzhi Yao	Jie Yao	Chunyan Ge
Hongfang Ju	Zhong Hua	Deming Ma	Yan Chen
Hua Qian	Tong Sun	Yu Zhang	Ren Qiang
Chunyan Ye	Yunchuan Pu	Lina Zhang	Aimin Cui
Defang Zhai	Zheng Wang	Xiaoying Yao	Jinxia Xu
Xueming Zhang	Qien Yang	Rong Zhang	Sue Jiang
Jianhong Pei	Xinguo Wang	Xuebing Yan	Wen Xu
Zheng Wei	Xiaoxia Tang	Xiucheng Pan	Yuxi Ma
Jianhua Jiang	Jianchun Xian	Ming Chen	Fangzheng Han
Hui Zhang	Zhongqin Wang		
**Jiangxi Province**			
Guojun Shen	Yun Luo	Min Kong	Peihua Yang
Ming Li	Ningning Wang	Guilian Zhong	Jingke Zeng
Lu Shao	Min Yang	Li Rui	Lijuan Long
Yilei Tao	Xiaolin Zhang	Xin Huang	Yuling Lan
Yushan Lu	Shiqiong Zhou	Yinbai Fan	Xia Wang
Lifeng Cao	Liying Zhou	Xiaoxiong Hu	Wangui Zhang
Yun Hu	Yuan Fang	Meiling Huang	Dixiu Liu
Jing Zhang	Tao Yue	Manlei Jiang	Guanlin Zhou
Yao Wen	Fenglin Kong	Fei Xu	Xiaolan Wang

Shengping Fang			
**Liaoning Province**			
Yang Ding	Danyang Liu	Shu Sun	Lilan Shi
Xiaoguang Dou	Qiong Liu	Fang Zhan	Jingyan Wang
Qiuju Sheng	Qingwei Gao	Yan Wang	Lin Zhang
Chong Qiao	Xin Liu	Lin Bi	Han Bai
Jun Wei	Yanshan Liu	Baijun Li	Jun Ran
Zhuo Feng	Fengmin Xu	Fen Huang	Yong Wang
Bin Ning	Shijie Lyu	Xuelian Wang	Ping An
**Inner Mongolia**			
Zhongsheng Liu	Yan Zhang	Jinlian Zhou	Huiting Wang
Shuyi Suo	Guirui Bai	Meiying Shi	Haiyan He
Huanhuan Liu	Hong Xing	Guiying Nie	Na Ta
Min Li	Lulin Wang	Xinyue Wang	Huiyun Xu
Feiyun Bai	Hong Fan		
**The Ningxia Hui Autonomous Region**			
Xiangchun Ding			
**Qinghai Province**			
Hongmei Zu	Shengrong Zhang	Yu Zhang	Xiaoyan Zhang
Qinghua Lu	Junning Peng	Jiaying Yan	Hongmei Duo
Haifang Cao	Hude Wang		
**Shandong Province**			
Feng Gao	Suping Zhang	Xia Cui	Lu Guo
Qingyan Li	Hongxia Wang	Jianguo Yuan	Ru Chen
Xiaoge Yang	Ronghua Liu	Hongkui Zhao	Wenmei Chen
Xia Li	Ruxiu Xu	Qingfeng Shi	Lin Zhang
Xuejun Liu	Yanyun Wang	Zhenzhou Xu	Xiuping Yan
Lin Li	Tian Shao	Mei Zhang	Fenghua Liu
Xiumei Chen	Yali Cheng	Xin Wei	Xiaonuo Gao
Hong Wei	Yujun Liu	Wei Lu	Xia Li
Ruili Mou	Yunguang Li	Zhanjie Niu	Li Lin
Min Song	Qi Chen	Xue Li	Xin Geng
Xiaoping Li	Qingping Gao	Huanrong Feng	Yan Wang
Cuilan Wang	Jing Du	Linlin Sun	Qiaozhen Chen
Dong Hao	Sikui Wang	Shuxia Ge	Hui Lyu
Tingting Zheng	Shengru Zhang	Linuo Peng	Jilan Yu
Caixia Chen	Yuanzheng Gao	Xiaobing Wang	Jing Du
Guoguie Guo	Qingfang Li	Tong Yuan	Jie Li
Jian Li	Shan Guan	Rendong Wei	Huibin Zhu
Yuan Wu	Wei Chen	Jingjing Guo	Qinge Gao
Cuizhi Li	Peng Ning		
**Shanxi Province**			
Xihong Wang	Bo Wang	Jianmin Rong	Cuipeng Feng

Xiang Ma	Rui Li	Haiyan Zhu	Caihong Wu
Xiaoli Liang	Yiqun Qu	Xining Wang	
**Shaanxi Province**			
Tianyan Chen	Jiuping Wang	Yanjun Li	Jiejing Xin
Yingren Zhao	Ruifeng Tian	Tai Wang	Chunxia Li
Jinfeng Liu	Yu Liu	Jihong Feng	Bing Dong
Yuan Yang	Wei Zhang	Na Liu	Yutao Liu
Yingli He	Yage Zhu	Peidong Zhao	Chunyan Li
Taotao Yan	Feng Ding	Junxiao Qu	Pingping Zhang
Zhen Tian	Dan Liu	Yafang Zhang	Yuan Zhao
Dandan Guo	Guanghua Xu	Huaiqiang Pan	Yidan Zhang
Rongfang Xu	Xiaohong Gao	Chengfu Wang	Le Yao
Zhigang Liu			
**Shanghai**			
Lihui Jiang	Jun Zhao	Rui Guan	Junyao Lu
Li Yan	Chengzhong Li	Jinfeng Zeng	Lei Yan
Zhimin Han	Xuesong Liang	Peiru Jiang	Yupan Bai
Min Liu	Jianya Xue	Hongmei Deng	Yuanyuan Zhou
Xiaohong Zhang	Zhihui Chen	Jinghua Liu	Yaoyue Kang
Jielian Yang	Ruiying Zheng	Jie Xu	Yue Li
Yangqiu Chen	Jixiu Chen	Qin Fan	Hongjuan Chai
Minmin Sheng	Wei Yin	Xiaoling Yuan	Yunhui Zhuo
Jie Yang	Yuhuan Liu	Shengzhen Hong	Bei Luo
Weiwei Sun	Yu Chen	Donglin Yin	Chuanlou Xu
Jiayan Gu	Lin Zhou	Xue Yang	Lihong Qu
Fengdi Zhang	Yingqiu Shen		
**Sichuan Province**			
Min Zhou	Enqiang Chen	Xiaomei Zhong	Xiaoxia Geng
Kejing He	Lingyao Du	Yi Zeng	Xuebing Chen
Huachun Yin	Libo Yan	Li Yin	Jiahong Yang
Juan Li	Hong Li	Qijun Cheng	Feifei Liu
Zhaohui Zhu	Juan Tang	Limin Zhou	Wanrong Luo
Mingxiang Wu	Chunfang You	Tingting Luo	Bibo Wu
Yufen Li	Jing Tang	Jianmei Lin	Fuli Shu
Haixia Huang	Wei Deng	Xingxiang Yang	Shushu Liu
Lang Bai	Yijun Liu	Renguo Yang	Han Zhuang
Hong Tang	Jianli Xu	Rengang Huang	Rong Hu
Shuqiang Wang	Xia Zhu		
**Tianjin**			
Hai Li	Xin Guo	Jinyu Hao	Huiying Yang
Shumin Ning	Jing Chen	Jing Hao	Yanli Shi
Yurong Zhang			
**Tibet**			

Li Shi	Deji Ciren	Qiongda Cidan	Bazhen Ciwang
Sang Ba	Panduo Dawa	Panduo Laba	Panduo Laba
Qingping Wen	Quwang Danzeng	Wenfan Luo	Quanyan Zhu
Lamu Mima	Lamu Bianba	Ciren Tudan	Daoping Han
Zhenzhen Wu			
**The Xinjiang Uyghur Autonomous Region**			
Qin Xu	Hongfeng Wang	Feng Guo	Zhuanguo Wang
Xiaozhong Wang	Huxibaiheti	Xiaobo Wang	Dan Han
Ka Ni	Yan Ma	Xiaofang Zhuang	Jie Zhang
Yonghong Yue	YanWang	Qiang Fu	
**Yunnan Province**			
Jing You	Hongli Zhang	Lu Zhang	Yilan Xia
Jinghua Fan	Junxin Zhang	Jiawei Geng	Ling Zhu
Guowei Li	Yihui Chen	Wei Yue	Xiaoqing Wang
Wu Li	Chunmei Chao	Yulong Wang	Xiao Liang
Hong Dai	Yanmei Zhang	Bing Bu	Xiuying Ma
Weibo Yang	Xianli Li	Liping Huang	Ruyi Zhang
Ying Niu	Ju Zhou		
**Zhejiang Province**			
Suying Zhang	Wanfeng Hu	Lingyan Shen	Chuantong Lu
Shourong Liu	Hongliang Ou	Hua Xuan	Yingming Fei
Xin Luo	Lingyan Fan	Xu Wang	Furong Liu
Zhe Yu	Xijie Lai	Danfeng Sun	Fuyan Sun
Chun Zhao	Chen Wang	Xiao Yu	Tao Xiong
Xiankai Wang	Xiaofeng Guo	Min Deng	Hongping Xuan
Pei Hu	Chengjing Tao	Jianming Wu	Jie Jin
Yaoren Hu	Li Tian	Fanchun Fu	Wenbao Huang
Airong Hu	Dongfang Ni	Xuzhen Lu	Xiaoxian Jiang
Xiunong Jiang	Xinsheng Xie	Qianqian Zhou	Lifei Yu
Wen Zhang	Xiong Sheng	Jieping Li	Jinfeng Shi
Jiong Wan	Yunqing Chen	Biao Zhu	Yi Jiang
Wenying Jiang	Hong Wang	Lin Qiu	Xiaoxiao Liu
Lingyun Zhang	Xiaofu Yang	Jie Wang	Chenwei Pan
Guoxian Zhu	Xiaoxia Bai	Yanting Bao	Aiqun Ren
Min Wu	Ran Ding	Suzhao Pan	Xiaobo Ying
Xiang Zhou	Deng Huang	Lifang Guo	Ligang Xu
Lidan Zhang	Wangwang Xu	Jiguang Ding	Yi Lin
Jing Zhu	Bingqi Ye	Qingwei Du	Gongying Chen
Huiqin Li	Tingting Pan	Danmin Wang	Yafen Qiu
Caixia Xia	Liqin Yu	Hongying Pan	Danhong Yang
**Chongqing**			
Wanju Yang	Fengying Wang	Xueyan Wang	Yi Wu
Wenling Cai	Qing Mao	Jie Xia	Wei Sun

Hua Hu	Ting Xie	Junnan Li	Chuwen Li
Qinghua Zhang	Xiaohong Wang	Lan Tao	

12. References

1. Ganem D, Prince AM. Hepatitis B virus infection-natural history and clinical consequence. *N Engl J Med.* **350**,1119-1129(2004).

2. Lavanchy D. Hepatitis B virus epidemiology, disease burden, treatment, and current and emerging prevention and control measures. *J Viral Hepat.* **11**,97-107(2004).

3. Liang X. et al. Epidemiological serosurvey of hepatitis B in China-declining HBV prevalence due to hepatitis B vaccination. *Vaccine* **27**, 6550-6557(2009).

4. Liang X. et al. Evaluation of the impact of hepatitis B vaccination among children bomduring 1992-2005 in China. *J Infect Dis*. **200**, 3947(2009).

5. Zou H, Chen Y, Duan Z, Zhang H, Pan C. Virologic factors associated with failure to passive-active immunoprophylaxis in infants born to HBsAg-positive mothers. *J Viral Hepat.***19**,e18-e25(2012).

6. Wu Q. et al. Telbivudine Prevents Vertical Transmission of Hepatitis B Virus From Women With High Viral Loads: A Prospective Long-Term Study. *Clin Gastroenterol Hepatol.* **13**,1170-1176(2014).

7. Han GR. et al. A prospective and open-label study for the efficacy and safety of telbivudine in pregnancy for the prevention of perinatal transmission of hepatitis B virus infection. *J Hepatol.* **55**,1215-1221(2011).

8. Pan CQ. et al. Telbivudine prevents vertical transmission from HBeAg-positive women with chronic hepatitis B. *Clin Gastroenterol Hepatol.* **10**,520-526(2012).

9. Xu WM. et al. Lamivudine in late pregnancy to prevent perinatal transmission of hepatitis B virus infection: a multicentre, randomized, double-blind, placebo-controlled study. *J Viral Hepat.***16**,94-103(2009).

10. Zhang H, Pan C, Pang Q, Tian R, Yan M, Liu X. Telbivudine or lamivudine use in late pregnancy safely reduces perinatal transmission of hepatitis B virus in real-life practice. *Hepatol.* **60**,468-476(2014).

11. European Association For The Study Of The Liver. EASL clinical practice

guidelines: Management of chronic hepatitis B virus infection. *J Hepatol*. **57**,167-185(2012).

12. Liaw YF. et al. Asian-Pacific consensus statement on the management of chronic hepatitis B: a 2012 update. *Hepatol Int.***6**,531-561(2012).
