# Supplementary material for: Real-world implementation of a multilevel interventions program to prevent mother-to-child transmission of HBV in China
Source: Nat Med. 2024 Jan 31;30(2):455–62. doi: 10.1038/s41591-023-02782-x (PMC10878969; doi:10.1038/s41591-023-02782-x)
Supplement: Supplementary file 2 — Reporting Summary [file 41591_2023_2782_MOESM2_ESM.pdf]

nature portfolio

Corresponding author(s): Jinlin Hou

Last updated by author(s): Dec 5, 2023

Reporting Summary

Nature Portfolio wishes to improve the reproducibility of the work that we publish. This form provides structure for consistency and transparency in reporting. For further information on Nature Portfolio policies, see our Editorial Policies and the Editorial Policy Checklist.

Statistics

For all statistical analyses, confirm that the following items are present in the figure legend, table legend, main text, or Methods section.

n/a	Confirmed	
☐	☒	The exact sample size (*n*) for each experimental group/condition, given as a discrete number and unit of measurement
☒	☐	A statement on whether measurements were taken from distinct samples or whether the same sample was measured repeatedly
☐	☒	The statistical test(s) used AND whether they are one- or two-sided<br>*Only common tests should be described solely by name; describe more complex techniques in the Methods section.*
☐	☒	A description of all covariates tested
☐	☒	A description of any assumptions or corrections, such as tests of normality and adjustment for multiple comparisons
☐	☒	A full description of the statistical parameters including central tendency (e.g. means) or other basic estimates (e.g. regression coefficient) AND variation (e.g. standard deviation) or associated estimates of uncertainty (e.g. confidence intervals)
☐	☒	For null hypothesis testing, the test statistic (e.g. *F*, *t*, *r*) with confidence intervals, effect sizes, degrees of freedom and *P* value noted<br>*Give P values as exact values whenever suitable.*
☒	☐	For Bayesian analysis, information on the choice of priors and Markov chain Monte Carlo settings
☐	☒	For hierarchical and complex designs, identification of the appropriate level for tests and full reporting of outcomes
☐	☒	Estimates of effect sizes (e.g. Cohen's *d*, Pearson's *r*), indicating how they were calculated

Our web collection on statistics for biologists contains articles on many of the points above.

Software and code

Policy information about availability of computer code

Data collection	A mobile health application called the "SHIELD APP" (version 15.7.21) was developed. During follow-up, all test reports were captured as pictures and uploaded into the SHIELD APP by participants. Additional information was uploaded into the SHIELD APP by the doctors or research assistants.
Data analysis	All data were analysed using R software 2022, version 4.2.2.

For manuscripts utilizing custom algorithms or software that are central to the research but not yet described in published literature, software must be made available to editors and reviewers. We strongly encourage code deposition in a community repository (e.g. GitHub). See the Nature Portfolio guidelines for submitting code & software for further information.

Data

Policy information about availability of data

All manuscripts must include a data availability statement. This statement should provide the following information, where applicable:

- Accession codes, unique identifiers, or web links for publicly available datasets
- A description of any restrictions on data availability
- For clinical datasets or third party data, please ensure that the statement adheres to our policy

The clinical data are not publicly availbale as the program is still on going. The data will be available upon reasonable request for academic use only. If other

investigators are interested in performing additional analysis, an application can be submitted to the corresponding author (jlhousmu@163.com), explaining the analyses planned. The request will be responded to within one month of receipt.

Research involving human participants, their data, or biological material

Policy information about studies with human participants or human data. See also policy information about sex, gender (identity/presentation), and sexual orientation and race, ethnicity and racism.

Reporting on sex and gender	In this study, the inclusion criteria were pregnant women who had been HBsAg positive for more than six months. Therefore, all participants were women.
Reporting on race, ethnicity, or other socially relevant groupings	This study did not involve any socially constructed or socially relevant categorization variable.
Population characteristics	A total of 30109 pregnant women from Shield program stage II and 8642 from stage III were included. The age was (28.36 ±4.33) year in stage II and (29.76±4.51) year in stage III A total of 53.51% of the mothers in stage II and 24.86% of the mothers in stage III had HBV DNA viral loads over 200,000 IU/mL, and 57.36% and 25.36% of the mothers were HBeAg positive, respectively.
Recruitment	The recruitment in stage II was convenience sampling in 178 hospitals nationwide. However, in stage III, all pregnant women with HBV infection from Baoan District were participated. Pregnant women who had been HBsAg-positive for over six months. Pregnant women were excluded if they had a positive serological test for HIV or HCV or had any comorbidity that could influence compliance. In our study, pregnant women who did not have a smartphone and did not want to use the app were excluded because we obtained data through the app.
Ethics oversight	This study was approved by the Nanfang Hospital ethics committee. Electronic informed consent was obtained via the SHIELD app.

Note that full information on the approval of the study protocol must also be provided in the manuscript.

Field-specific reporting

Please select the one below that is the best fit for your research. If you are not sure, read the appropriate sections before making your selection.

☒ Life sciences ☐ Behavioural & social sciences ☐ Ecological, evolutionary & environmental sciences

For a reference copy of the document with all sections, see nature.com/documents/nr-reporting-summary-flat.pdf

Life sciences study design

All studies must disclose on these points even when the disclosure is negative.

Sample size	A total of 30109 pregnant women from stage II and 8642 from stage III were included. The SHIELD program is still ongoing, so some participants are still under follow-up. We performed sample sizeestimation for the post-hoc analyses. The sample proportion is assumed to be 0.02. To produce a confidence interval with a width of no more than 0.005, 12047 subjects were needed. Assuming the dropout rate was 20%,15059 subjects will be needed (sample size was computed using PASS 2022, version 22.0.3.).
Data exclusions	In Stage II of Shield Program, 30,109 pregnant women were included in this analysis. 12 patients excluded for co-infection with HCV, and 23 patients excluded for any comorbidity that could influence compliance.
Replication	This is a real-world clinical study.
Randomization	This study is not a randomized, blinded clinical trial but a prospective cohort.
Blinding	The study was designed to be a prospective real world study. Given the ethics, it is not proper to design a randomized, blinded clinical trial.

Reporting for specific materials, systems and methods

We require information from authors about some types of materials, experimental systems and methods used in many studies. Here, indicate whether each material, system or method listed is relevant to your study. If you are not sure if a list item applies to your research, read the appropriate section before selecting a response.

Materials & experimental systems

n/a	Involved in the study
☒	☐ Antibodies
☒	☐ Eukaryotic cell lines
☒	☐ Palaeontology and archaeology
☒	☐ Animals and other organisms
☐	☒ Clinical data
☒	☐ Dual use research of concern
☒	☐ Plants

Methods

n/a	Involved in the study
☒	☐ ChIP-seq
☒	☐ Flow cytometry
☒	☐ MRI-based neuroimaging

Clinical data

Policy information about clinical studies

All manuscripts should comply with the ICMJE guidelines for publication of clinical research and a completed CONSORT checklist must be included with all submissions.

Clinical trial registration: No. NCT05172453

Study protocol: Protocol can be accessed in the supplementary materials

Data collection: This study was conducted in diverse health settings across China (30109 pregnant women from 178 hospitals in stage II: July 2015-January 2022 and 8642 pregnant women from 160 health facilities at community level in stage III: January 2018-December 2019).

Outcomes: The primary outcomes were the HBV MTCT rate and birth defect rate. HBV MTCT was defined as being seropositive for HBsAg of infants after completing PVST. Birth defects were diagnosed by the obstetricians referred to the national standards for medical diagnosis of birth defects.
